# Green synthesis of *Piper nigrum* copper-based nanoparticles: *in silico* study and ADMET analysis to assess their antioxidant, antibacterial, and cytotoxic effects

**DOI:** 10.3389/fchem.2023.1218588

**Published:** 2023-09-05

**Authors:** Modumudi Kiranmayee, Nambi Rajesh, M. Vidya Vani, Habeeb Khadri, Arifullah Mohammed, Suresh V. Chinni, Gobinath Ramachawolran, Khateef Riazunnisa, Ashaimaa Y. Moussa

**Affiliations:** ^1^ Department Biotechnology and Bioinformatics, Yogi Vemana University, Kadapa, India; ^2^ Department of Medical Laboratories, College of Applied Medical Sciences, Qassim University, Buraidah, Saudi Arabia; ^3^ Department of Agriculture Science, Faculty of Agro-Based Industry, Universiti Malaysia Kelantan, Jeli, Malaysia; ^4^ Department of Biochemistry, Faculty of Medicine, Bioscience, and Nursing, MAHSA University, Jenjarom, Malaysia; ^5^ Department of Periodontics, Saveetha Dental College and Hospitals, Saveetha Institute of Medical and Technical Sciences, Chennai, India; ^6^ Department of Foundation, RCSI & UCD Malaysia Campus, Georgetown, Malaysia; ^7^ Department of Pharmacognosy, Faculty of Pharmacy, Ain shams University, Cairo, Egypt

**Keywords:** *Piper nigrum*, *Piper nigrum* copper nanoparticles, cytotoxicity, molecular docking, alkamides, ADMET/TOPKAT, antibacterial activity, antioxidant activity

## Abstract

Nanobiotechnology is a popular branch of science that is gaining interest among scientists and researchers as it allows for the green manufacturing of nanoparticles by employing plants as reducing agents. This method is safe, cheap, reproducible, and eco-friendly. In this study, the therapeutic property of *Piper nigrum* fruit was mixed with the antibacterial activity of metallic copper to produce copper nanoparticles. The synthesis of copper nanoparticles was indicated by a color change from brown to blue. Physical characterization of *Piper nigrum* copper nanoparticles (PN-CuNPs) was performed using UV-vis spectroscopy, FT-IR, SEM, EDX, XRD, and Zeta analyzer. PN-CuNPs exhibited potential antioxidant, antibacterial, and cytotoxic activities. PN-CuNPs have shown concentration-dependent, enhanced free radical scavenging activity, reaching maximum values of 92%, 90%, and 86% with DPPH, H_2_O_2_, and PMA tests, respectively. The antibacterial zone of inhibition of PN-CuNPs was the highest against *Staphylococcus aureus* (23 mm) and the lowest against *Escherichia coli* (10 mm). PN-CuNPs showed 80% *in vitro* cytotoxicity against MCF-7 breast cancer cell lines. Furthermore, more than 50 components of *Piper nigrum* extract were selected and subjected to *in silico* molecular docking using the C-Docker protocol in the binding pockets of glutathione reductase, *E. coli* DNA gyrase topoisomerase II, and epidermal growth factor receptor (EGFR) tyrosine to discover their druggability. Pipercyclobutanamide A (26), pipernigramide F (32), and pipernigramide G (33) scored the highest Gibbs free energy at 50.489, 51.9306, and 58.615 kcal/mol, respectively. The ADMET/TOPKAT analysis confirmed the favorable pharmacokinetics, pharmacodynamics, and toxicity profiles of the three promising compounds. The present *in silico* analysis helps us to understand the possible mechanisms behind the antioxidant, antibacterial, and cytotoxic activities of CuNPs and recommends them as implicit inhibitors of selected proteins.

## Introduction

Nanotechnology emerged as an interdisciplinary approach with various applications. The emergence of nanoparticles (NPs) created a scientific revolution in terms of providing a sustainable environment for humans. NPs are promising due to their optical, catalytic, and surface-to-volume ratio (Singh et al., 2018; Sarma et al., 2019) and are currently employed to treat a variety of ailments, as well as for energy storage and drug/gene delivery systems (Pomerantseva et al., 2019; Fries et al., 2021).

Physical and chemical methods employed for the synthesis of NPs include lithography, ultrasonic fields, UV irradiation, and photochemical reduction (Mafuné et al., 2002; Naik et al., 2002). However, these methods require hazardous chemicals as reducing agents. Hence, green synthesis of nanoparticles was introduced as an alternative method that used either microbes or plant extract of medicinal plants as reducing agents. Green synthesis of nanoparticles by microbes is a time-consuming process (Thakkar et al., 2010) for maintenance, but a plant-extract-mediated process requires less time. Green synthesis of copper nanoparticles (CuNPs) using plants has been reported recently with *Cocculus hirsutus* (Ameena et al., 2022), *Kigelia africana* fruit, and *Sesbania aculeata* (Tamil Elakkiya et al., 2022). Natural compounds can act as an alternative for novel drug synthesis, and plant-derived compounds showed high efficiency and easy availability (Cragg and Newman, 2013). Medicinal herbs have various bioactive molecules exhibiting various biological properties, such as antioxidant, antimicrobial (Mseddi et al., 2020), antibiofilm (Gad-Elkareem et al., 2019), anti-quorum sensing (Snoussi et al., 2016), and antidiabetic (Moussa et al., 2013; Noumi et al., 2018) properties.

Gold, silver, platinum, ruthenium, zinc, and copper are various metals used for the synthesis of metal NPs (Akintelu et al., 2019; Akintelu and Folorunso, 2019; Folorunso et al., 2019; Nasrollahzadeh et al., 2019; Almatroudi et al., 2020; Anjum and Riazunnisa, 2022). Among these metal NPs, copper nanoparticles (CuNPs) are gaining popularity because of their high surface area, low cost, and eco-friendliness. Physical characteristics of CuNPs such as morphology, crystallinity, composition (Cuevas et al., 2015), a simple methodology for synthesis, and easy modification into the required shape and size make them exceptional (Balasooriya et al., 2017). The biological activities of CuNPs make them an ideal source for antibiotic production, as new antibiotics are urgently needed (Chung et al., 2017; Moussa et al., 2020). According to the WHO, 177 ± 16 ppb is the permissible limit of copper (Sudha et al., 2012) in stored food and drinking water. Copper vessels purify drinking water by killing *Escherichia coli*, rapidly and efficiently destroying bacteria through a contact-killing mode (Santo et al., 2011).

Applying *in silico* molecular docking to augment drug discovery research has gained much scientific interest in recent years. Not only does it save time and effort spent on inactive molecules, but also resources are spared to develop promising drug entities to clinical stages. Virtual screening allows the use of chemical structures of literature compounds compiled from eminent databases, filtered, and prepared to adopt a proper experimental design to predict the activity inside the binding pockets of certain enzymes (Altyar et al., 2022). *Piper nigrum*, also known as pepper, is a member of the Piperaceae family. It is known as the “king of spices” (Takooree et al., 2019). *P. nigrum* seed is used as an acceptable food additive (Meghwal and Goswami, 2013). Piperine, an alkaloid responsible for the pungent odor, is used in traditional medicine and as an insecticide; it also has anticonvulsant properties. Phytochemicals present in black pepper include phenols, flavonoids, alkaloids, amides, essential oils, lignans, neolignans, terpenes, and chalcones (Liu et al., 2010; Shityakov et al., 2019).

The water extract obtained from *P. nigrum* fruits was shown to be rich in pungent principles such as alkamides and piperine alkaloids; thus, these compounds were assembled from the literature to perform virtual screening. The same crude extract was utilized in all the bioassays described herein. The literature search revealed more than 55 compounds isolated from *P. nigrum* fruits along with volatile metabolites. Due to the presence of biologically active components, they are considered ideal natural therapeutic agents against bacterial, cancer, and fungal cells (Aldaly, 2010; Reshmi et al., 2010; Zhang et al., 2021). Various bio-activities were correlated with the alkaloid/alkamide components, particularly, piperine (Tyagi et al., 2021). While 1% of *P. nigrum* fruits comprised essential oils, dominated by *α*-pinene, gluulol, *α*-terpinene, and *β*-caryophyllene, 5%–9% represented pungent principle alkamides. In this study, approximately 60 alkamide/alkaloid compounds and their derivatives were selected to investigate their binding properties and potential as antioxidants, antibacterial, and antitumor agents. Furthermore, ADMET/TOPKAT analysis was conducted to reveal their pharmacokinetic properties, pharmacodynamics, and toxicity.

**FIGURE 1:**
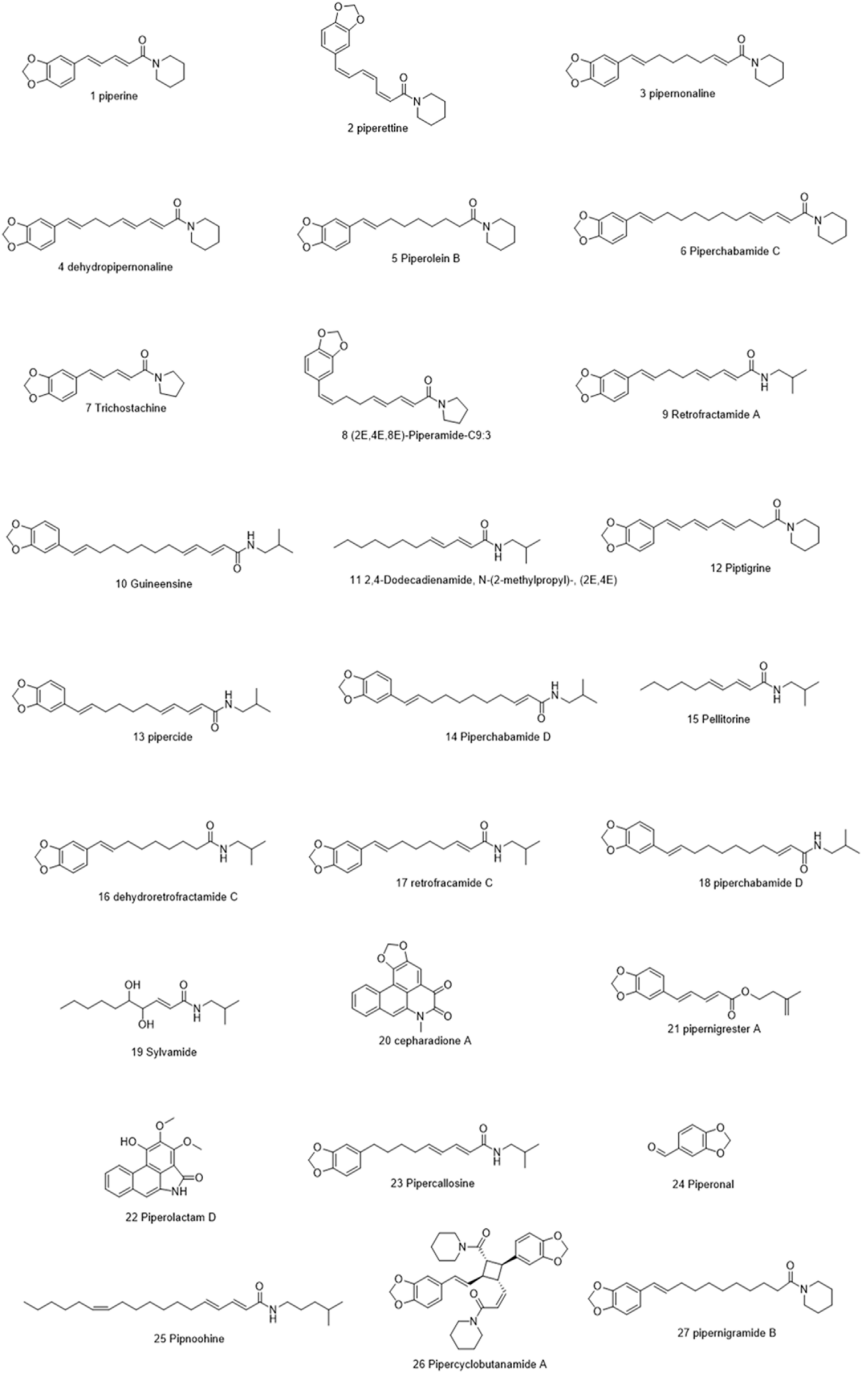
Chemical structures of some selected alkamides in *Piper nigrum* fruits.

**FIGURE 2:**
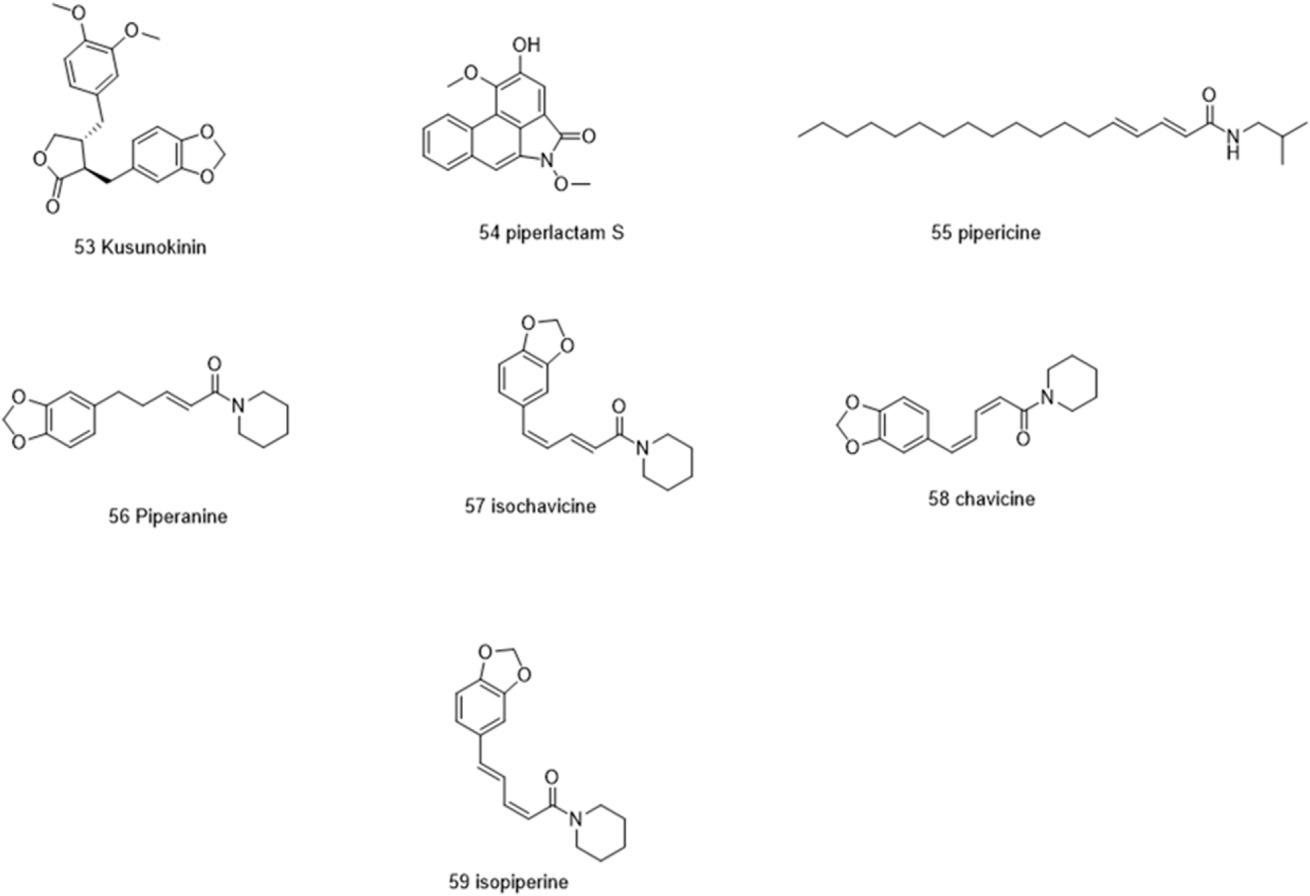
Chemical structures of some selected alkamides in *Piper nigrum* fruits.

**FIGURE 3:**
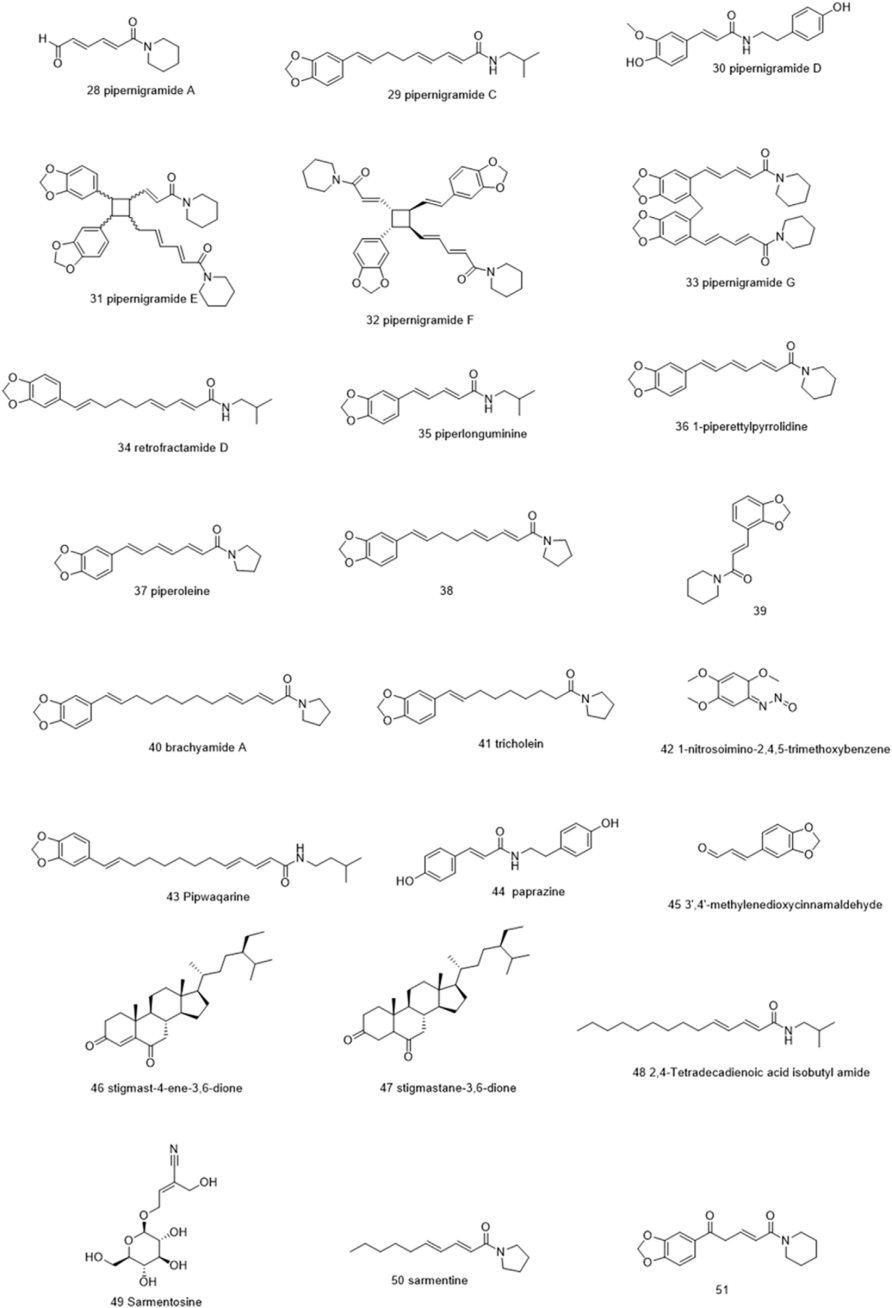
Chemical structures of some selected alkamides in *Piper nigrum* fruits.

Molecules selected for the *in silico* study are as follows: the active chemosensate components in black pepper, sharing piperidine, pyrrolidine, and isobutyl amide group, and classified as two categories of amides: the piperonal moiety-associated amides and the long chain fatty acid unsaturated amides (Dawid et al., 2012). The anti-inflammatory alkamides, including pipernonaline, guineensine, pellitorine, retrofractamide C, piperolein B, (2E,4Z,8E)-N-[9-(3,4-methylenedi-oxyphenyl)-2,4,8-nonatrienoyl]piperidine, piperchabamide D, dehydropipernonaline, retrofractamides, and pipernigramides, are also investigated (Figures 1–3) (Scott et al., 2005; Rho et al., 2007; Yun et al., 2018; Shityakov et al., 2019; Barata et al., 2021).

Piperine, the well-known alkaloid isolated from *P. nigrum* hundreds of years ago, showed the lowest threshold of pungency followed by piperyline and piperettine. Indeed, the isobutyl amine- and pyrrolidine-containing compounds revealed a higher threshold than any of the piperidine analogs (Dawid et al., 2012) Some of these compounds, such as piperonylamine, pipericide, sarmentosine, sarmentine, and chavicine, are bioactive compounds with pharmacological effects (Figure 1) (Majdalawieh and Carr, 2010). In particular, piperine is believed to be the main bioactive chemical component with antimicrobial activities purified from *P. nigrum* (Mohammed et al., 2016).

Bacterial antibiotic resistance will result in serious human health problems and deaths. If this issue is not taken seriously, approximately 10 million people will die from antimicrobial-resistant bacterial infections by 2050. Recently, nanotechnology-based strategies have attracted attention to the development of new diagnostic technologies and devices in modern medicine. CuNPs have been extensively used in the medical field due to their superb antimicrobial nature against multidrug-resistant pathogens.

Phytochemicals present in *P. nigrum* fruit extract might be involved in the reduction and stabilization of CuNP synthesis. Therefore, the current study focuses on the green synthesis of *P. nigrum* fruit-based copper nanoparticles using aqueous *P. nigrum* fruit extract, as well as their physical and biological characterization (antioxidant, antibacterial, and *in vitro* cytotoxicity). The invention of the unique size, shape, and structure of CuNPs exhibits multispectrum activity and has the potential to obstruct bacterial, cancer growth, and infection prevalence in clinical applications. Furthermore, we evaluated these *in vitro* activities with *in silico* studies to determine the correlation between them. Molecular docking studies were performed on the key active compounds in black pepper to manifest their druggability in the active sites of epidermal growth factor receptor (EGFR), DNA gyrase topoisomerase II, and glutathione reductase.

## Materials and methods

### *Piper nigrum* fruit collection and preparation of fruit extract

*P. nigrum* fruits were purchased from the local market in Kadapa, Andhra Pradesh, India. India is the major producer of *P. nigrum.* A voucher specimen of *P. nigrum* fruit (from the sample collected from a local market in Kadapa, Andhra Pradesh) was deposited and authenticated by Dr. A. Madhusudhana Reddy, Head of Herbarium Division, Dept. of Botany, Yogi Vemana University, Kadapa, India (the voucher specimen number of *Piper nigrum* fruit is YVU/KR-5301-5302).

*P. nigrum* fruits were washed, shade-dried, and powdered before being used. A measure of 10 g of powdered *P. nigrum* was mixed with 100 mL of double-distilled water and boiled in a water bath at 70–80°C for 15–20 min. The solution was cooled and filtered using Whatman No: 1 filter paper. The filtrate solution was then stored at **4** **C** for further analysis (Tyagi et al., 2021).

### Synthesis of *P. nigrum* fruit extract copper nanoparticles

*P. nigrum* copper nanoparticles (PN-CuNPs) were synthesized by adding 10 mL of fruit extract dropwise to 200 mL of copper acetate (3 mM) solution on a magnetic stirrer. The color change of the solution from brown to blue represents the formation of PN-CuNPs. The reduction of copper ions was monitored using a UV-visible spectrometer (Thermo Scientific Evolution 201) with a wavelength range of 200–800 nm. The reaction mixture was centrifuged at 10,000 rpm for 10 min, and the pellet was washed with distilled water and air-dried. The synthesized PN-CuNPs were stored at 4°C for further analysis.

## Characterization of PN-CuNPs

### UV-vis spectrophotometry analysis

The absorption maxima of 2 mL of freshly prepared reaction mixture of PN-CuNPs were obtained using a UV-Vis spectrophotometer (Thermo Scientific Evolution 401). The spectra were recorded with a wavelength range of 200–800 nm at regular time intervals (10, 15, 30, 45, 30, 45, 60, 120, and 180 min) using double-distilled water as a blank. We calculated the band gap energy using the UV spectrum data following Sabouri et al. [74].

### X-ray diffraction analysis of PN-CuNPs

X-ray diffraction (XRD) analysis of PN-CuNPs was performed on a PANLytical ‘X’ Pert Pro diffractometer operated at 40 kV and 30 mA with Cu K-alpha radiation. The average size of PN-CuNPs can be calculated using the Debye–Scherer equation as follows:

D=Kλ / β⁡Cosθ, (1)

where D is the crystallite size of the copper nanoparticles, *λ* is the wavelength, β denotes the full width at half maximum of the diffraction peak, k denotes the Scherer constant with a value from 0.9–1, and θ denotes the Bragg angle.

### FT-IR analysis of PN-CuNPs

Dehydrated PN-CuNPs and *P. nigrum* fruit extract were used to analyze functional groups. A measure of 10 mg of PN-CuNPs and *P. nigrum* fruit extract was mixed with 100 mg of KBr, vigorously ground into a fine powder, and compressed into diaphanous pellet discs followed by FT-IR [PerkinElmer (Spectrum Two model), UK VERTEX 70 model, Bruker, Germany]. Chemical and functional groups of the samples were obtained in the range of 400–4000 cm^−1^ at room temperature. The probable biomolecules responsible for the reduction, capping, and effective stabilization of the PN-CuNPs were recorded using an FT-IR spectrophotometer (Kanniah et al., 2021).

### SEM and EDAX analysis of PN-CuNPs

The morphology and chemical composition of PN-CuNPs were examined by scanning electron microscopy (Model: EVO 18; Carl Zeiss, Germany) equipped with an energy-dispersive X-ray spectrometer (EDX). EDX was performed at an acceleration voltage of 20–40 keV. Thin films of PN-CuNPs were placed on a gold-coated grid by sprinkling a very small amount of PN-CuNPs on the grid. Thin films on the SEM grid were dried for 5 min and examined at different angles.

### Particle size and zeta potential determination

The zeta potential and size of PN-CuNPs were measured by a Zetasizer Nano series instrument between 0.1 and 10,000 nm (Malvern Nano-ZS90). The stability of the nanoparticle sample was measured by maintaining the temperature of 25°C dispersion, 0.894 mPa of dispersion medium viscosity, and 0.073 mS/cm conductivity with 3.8 V electrode voltages.

### *In vitro* antioxidant assays

#### DPPH free radical scavenging assay of *P. nigrum* fruit extract and PN-CuNPs

*P. nigrum* fruit extract and biosynthesized PN-CuNPs were subjected to 2,2-diphenyl-1-picrylhydrazyl (DPPH) analysis. Different concentrations of PN-CuNPs (25–150 μg/mL) and fruit extract were mixed with 3 mL of methanolic solution containing DPPH radicals (0.1 mM). Ascorbic acid was taken as a positive control. After 30 min, the absorbance was determined at 517 nm and converted into % using the following formula:

$$\%\;of\;inhibition=\left[A_{0}-A_{1}/A_{0}\right]\times 100,$$ (2)

where A_0_ = absorbance of the control and A1 = absorbance of the test.

#### Hydrogen peroxide scavenging assay of *P. nigrum* fruit extract and PN-CuNPs

A modified protocol (Pavithra and Vadivukkarasi, 2015) was used for the H_2_O_2_ scavenging assay. Different concentrations of PN-CuNPs (25–150 μg/mL) and *P. nigrum* fruit extract were mixed with 40 mM H_2_O_2_ solution and allowed to incubate for 10 min, and the absorbance was measured at 230 nm. A positive control of ascorbic acid was used.

$$\%\;of\;scavenging\;\left(H_{2}O_{2}\right)=\left[A_{0}-A_{1}/A_{0}\right]\times 100,$$ (3)

where A_0_ = absorbance of the control, and A_1_ = absorbance of the test.

#### Phosphomolybdenum assay of *P. nigrum* fruit extract and PN-CuNPs

A modified protocol (Pavithra and Vadivukkarasi, 2015) was used to estimate total antioxidant activity. Molybdate reagent solution was prepared by mixing 0.6 M sulfuric acid, 28 mM sodium phosphate, and 4 mM ammonium molybdate. Different concentrations of PN-CuNPs (25–150 μg/mL) and *P. nigrum* fruit extract were added to each test tube, which individually contained 3 mL of distilled water and 1 mL of molybdate reagent solution, followed by incubation in a water bath at 95°C for 90 min. After incubation, these test tubes were cooled to room temperature for 20–30 min, and the absorbance was measured at 695 nm using ascorbic acid as a positive control.

#### Antibacterial activity of *P. nigrum* fruit extract and PN-CuNPs

The antibacterial activity of PN-CuNPs and *P. nigrum* fruit extract was examined using the agar well method. *Staphylococcus aureus* and *Bacillus subtilis* (Gram-positive) and *Escherichia coli* and *Proteus vulgaris* (Gram-negative) were chosen to investigate the antibacterial efficacy of PN-CuNPs and *P. nigrum* fruit extract. Different concentrations of PN-CuNPs (25, 50, 75, 100, and 150 μg/mL) and *P. nigrum* fruit extract were used along with positive control ampicillin. Plates were incubated at 37°C for 24 h, and the diameter of the antibacterial zone of inhibition was measured.

#### Cytotoxicity of *P. nigrum* fruit extract and PN-CuNPs

The cytotoxicity of synthesized PN-CuNPs and *P. nigrum* fruit extract against MCF-7 cells was measured by MTT assay (cell lines were purchased from the National Centre for Cell Science (NCCS), Pune, India). MCF-7 cells were seeded at a density of 5 × 10^4^ cells/well in 96-well plates, then treated with various concentrations of PN-CuNPs (25–200 μg/mL) and *P. nigrum* fruit extract, and incubated at 37°C for 24 h at 5% CO_2_ and 95% humidity. The cells were treated with MTT (5 mg/mL), and the absorbance was recorded at 570 nm.

#### *In silico* molecular docking studies of *P. nigrum* bioactive compounds with EGFR, DNA gyrase, and glutathione reductase

A total of 58 compounds were selected from the literature as the most bioactive components of *P. nigrum* fruits, specifically, alkamide/alkaloids. The molecular docking was designed to examine the binding sites of three proteins: glutathione reductase (Pdb ID: 1Xan), DNA gyrase (Pdb ID: 1Kzn), and EGFR (Pdb ID: 1m17). The X-ray crystal structures were downloaded from the Protein Data Bank (PDB) website (https://www.rcsb.org/), with their binding native ligands, HXP xanthene, clorobiocin, and erlotinib, respectively. The 3D chemical structures of the tested molecules and native ligands were prepared, and their energy was minimized by the MMFF94x force field using our previous protocol (Moussa et al., 2020). The computational study was performed using Discovery Studio 4.5 software at Ain Shams University, Faculty of Pharmacy, where ligands and enzymes were prepared using the molecular docking C-Docker protocols (Abdel Razek et al., 2020; Moussa et al., 2020; Ibrahim and Moussa, 2021).

By employing the pH ionization mode to 7.5, the physiological state is best matched and simulated with respect to the types of conformers, isomers, or tautomers produced; accordingly, the ligand’s Lipinski filter is adjusted. The root mean square deviation (RMSD) value was calculated for the docking poses and found to be 1.0, which proved the accuracy of the reproduced poses and validated the docking process. This was accomplished by removing the co-crystallized ligand from the active site (experimental) and superimposing with the docked native ligand (calculated).

The *in vitro* standards ampicillin, ascorbic acid, and doxorubicin were docked in their respective binding pockets to better assess and compare the significance of the ligands’ binding interactions and free energy. After docking, visualization was conducted using Discovery Studio Visualizer 2022, and the top 10 poses were selected for each tested molecule according to the Gibbs free energy. Preferences were given between molecules according to their interaction similarity, covalent and non-covalent, with the binding ligands and standards. The binding Gibbs free energy was obtained from the following formula and as per an implicit solvation model to present the best poses:

$$∆Gbinding=Ecomplex-\left(Eprotein+Eligand\right).$$ (4)

Here, ∆Gbinding denotes the binding energy of the ligand–protein interaction.

Ecomplex denotes the potential energy for the complex of the ligand bound with the protein.

Eprotein denotes only the protein potential energy.

Eligand denotes only the ligand potential energy.

##### Molecular docking simulations

The Glide docking wizard embedded in Maestro was employed to filter ligands and measure their receptor-free binding energy according to the extra precision criteria (XP). The docking score generated (Glide XP docking score) is calculated via an “XP Pose Rank” property using the following equation that takes into account the E-model and the Glide score:

Docking score (Glide score) = a*vdW + b*Coul + Hbond + Lipo + Metal + RotB + site.

The default settings adjusted in the grid generation were the OPLS 2005 force field, the van der Waals factor (1.00), and a 0.25 control factor for the workload. The grid box dimensions utilized were 14 Å × 14 Å × 14 Å, which are sufficient for ligand fitting. The best poses were assigned according to the lowest Gibbs free energy, RMSD values, and favorable orientation in the binding pockets. System validation was carried out by re-docking the two co-crystallized ligands into their primary protein pockets to measure the RMSD values, which were revealed to be 0.0301, 0.0041, and 0.6626 for 1m17, 1Kzn, and 1Xan, respectively. The values of RMSD less than 2 between the reference ligands before and after the molecular docking indicated the accuracy and precision of the docking protocols used.

## Results and discussion

### Synthesis of PN-CuNPs

The color change from brown to blue validated the synthesis of PN-CuNPs. This was mediated by *P. nigrum* fruit extract, which is involved in the reduction of Cu^+^ to Cu^0^. UV-visible spectral analysis revealed a strong peak at 500 nm due to surface plasmon resonance (Supplementary Figure S1B) (Ghosh et al., 2020; Mali et al., 2020; Sreeja et al., 2020). Secondary metabolites such as alkaloids, essential oils, polyphenolic compounds, and terpenoids act as stabilizing and capping agents and may be responsible for the reduction of Cu^+^ to Cu^0^ (Ghosh et al., 2020).

## Characterization of PN-CuNPs

### UV-vis spectrophotometer analysis of PN-CuNPs

The copper solution turned from brown to blue color after the addition of *P. nigrum* fruit extract powder, which indicated the synthesis of PN-CuNPs (Supplementary Figure S1B). The color change occurred due to the surface plasmon resonance of CuNPs (Mali et al., 2020; Sreeja et al., 2020). Supplementary Figure S1C shows that the optical band gap was calculated using the Tauc relation by plotting (ahn)2 vs. hn and extrapolating the linear portion of the curve to (ahn)2 ¼ 0. Hence, the optical energy band gaps of the PN-CuNPs were 3.7 eV. The band gap energy reveals that the PN-CuNPs have semiconductor properties.

### X-ray diffraction analysis of PN-CuNPs

The crystalline nature of the synthesized PN-CuNPs was characterized by XRD analysis and is presented in Supplementary Figure S2. The signals at different 2 theta values associated with the particular designs support the production of copper nanoparticles. For instance, the planes (11–1), (111), (20–2), (202), (−113), (31–1), (220), (221), and (22–2) are indexed for the peaks at 2 h values of 35.46, 38.62, 48.78, 53.58, 59.10, 61.42, 66.14, 67.86, and 74.93. These peaks match those in the ICDD PDF card no. 96-901-6327 standard database. The average size of PN-CuNPs crystal created through green synthesis was approximately 14 nm calculated using the Debye–Scherer equation. The aforementioned results are consistent with Amer and Awwad (2020) and Ghosh et al. (2020).

### FT-IR analysis of PN-CuNPs

FT-IR data of *P. nigrum* extract and PN-CuNPs represent probable functional groups of phytochemicals involved in bio-reduction as well as stabilization of PN-CuNPs (Supplementary Figure S3; Supplementary Table S1). The 3801 cm^−1^ absorption band of PN-CuNPs corresponds to the O-H stretch of alcohol. The absorption band of PN-CuNPs at 3377 cm^−1^ is due to N-H stretching, which is due to aliphatic primary amine functional groups. The 1,522 cm^−1^ band corresponds to C-H and C=C functional groups. The peaks of both extract and NPs are represented in Supplementary Table S1. Some of the peaks decreased in intensity, and some of the peaks shifted in their position. The decrease in the intensities and absorbance frequencies of the peaks at 3377, 2929, and 2854 cm^−1^ indicate the involvement of amines and alkane groups (N-H and C-H stretch) in the bio-reduction of NPs. PN-CuNPs exhibit an asymmetric stretching absorption band of 1408 cm^−1^. Phytochemicals present in *P. nigrum*, such as alkaloids, essential oils, polyphenolic compounds, and terpenoids, were involved in the bio-reduction of Cu^+^ into PN-CuNPs (Kanniah et al., 2021; Bawazeer et al., 2022).

### SEM and EDX spectral analysis of PN-CuNPs

The external morphology of PN-CuNPs, which form a spindle-shaped structure, was observed through SEM (Supplementary Figure S4A). Elemental analysis of PN-CuNPs was investigated by energy-dispersive X-ray analysis (Supplementary Figure S4B) at 1 and 8 keV. It displays the percent and composition of Cu metal as a significant peak, and other small peaks correspond to biomolecules capping the PN-CuNPs. Supplementary Figure S4B illustrates the percentages of Cu and other elements.

### Particle size and zeta potential determination of PN-CuNPs

The hydrodynamic size and surface charge of PN-CuNPs were revealed by a particle size analyzer and zeta potential analyzer. The average diameter of PN-CuNPs was 30–32 nm with negative surface zeta potential (−50 mV), and it remained constant over a period confirming the stability and the strong repulsion among the particles (Supplementary Figures S5A, B). This observation can be attributed to the presence of biomolecules as stabilizing agents in the leaf extract. The zeta potential measures the surface charge of particles. As the zeta potential increases, the surface charge of the particles increases. The zeta potential greatly influences particle stability in suspension through electrostatic repulsion between particles. Our results are consistent with Majdalawieh and Carr (2010) and Mohammed et al. (2016).

### *In vitro* antioxidant assays

#### Free radical scavenging assay of *P. nigrum* fruit extract and PN-CuNPs

PN-CuNPs and *P. nigrum* fruit extracts were subjected to DPPH assay. PN-CuNPs showed an elevated DPPH free radical scavenging activity compared to *P. nigrum* fruit extracts. A higher concentration of PN-CuNPs resulted in more free radical scavenging activity. PN-CuNPs showed the highest inhibition, approximately 92%, at a concentration of 150 μg/mL (Supplementary Figure S6) (Nahak and Sahu, 2011; Alminderej et al., 2021).

#### Hydrogen peroxide scavenging assay of *P. nigrum* fruit extract and PN-CuNPs

PN-CuNPs and *P. nigrum* fruit extract were examined for their H_2_O_2_ free radical scavenging activity. PN-CuNPs exhibited a higher rate of H_2_O_2_ free radical scavenging activity than *P*. *nigrum* fruit extracts. Increasing the concentration of PN-CuNPs yielded more free radical scavenging activity. PN-CuNPs showed the highest inhibition, approximately 90%, at a concentration of 150 μg/mL (Supplementary Figure S6B).

#### Total antioxidant activity of *P. nigrum* fruit extract and PN-CuNPs

The total antioxidant activity (TAA) of PN-CuNPs and *P. nigrum* fruit extracts was determined by the phosphomolybdenum method. PN-CuNPs exhibited an increased free radical scavenging activity compared to *P. nigrum* fruit extracts. Increased concentration of PN-CuNPs resulted in a higher TAA. The TAA of PN-CuNPs ranged from 68% to 86%. PN-CuNPs showed the highest amount of TAA, 86%, at a concentration of 150 μg/mL (Supplementary Figures S6C, S7). CuNPs synthesized from *Cocculus hirsutus* leaf extract also exhibited significant free radical scavenging activity (Ameena et al., 2022).

#### Antibacterial activity of *P. nigrum* fruit extract and PN-CuNPs

PN-CuNPs and *P. nigrum* fruit extracts were tested for antibacterial activity against human pathogenic microbes such as *S. aureus* (Gram-positive), *B. subtilis* (Gram-positive), *E. coli* (Gram-negative), and *P*. *vulgaris* (Gram-negative) by the agar well diffusion method. PN-CuNPs showed the highest antibacterial zone of inhibition with increasing concentration. The antibacterial zone of inhibition was in the range of 10 ± 0.17 mm to 23 ± 0.52 mm (Supplementary Figures S8A–D, S9). *S. aureus* exhibited the maximum antibacterial zone of inhibition of approximately 23 ± 0.52 mm (Supplementary Figure S8A) at a 100 μg/mL concentration, and the minimum zone of inhibition was found with *E. coli* (10 ± 0.17 mm) at 25 μg/ml, as depicted in Supplementary Figure S8C. Therefore, *S. aureus* was more susceptible to PN-CuNPs (Shah et al., 2019; Alminderej et al., 2021).

#### Proposed mechanism of antibacterial activity of PN-CuNPs

PN-CuNPs exhibited a larger antibacterial zone of inhibition against Gram-positive bacteria such as *S. aureus* than against Gram-negative bacteria. Copper is an essential metal for the growth of microbes at lower concentrations, but at higher concentrations, it will inhibit the growth of bacteria by penetrating the bacterial cell wall. PN-CuNPs penetrate the bacterial cell wall and stop the growth of bacteria. The size and shape of CuNPs influence their antibacterial activity. The free surface energy of the particles changes with size and morphology, as does the pH inside the cells (Karlsson et al., 2015). The proposed mechanism of action of antibacterial activity of PN-CuNPs is depicted in Supplementary Figure S10. The mechanism of action of PN-CuNPs occurs by enzyme interaction with –SH groups leading to DNA damage and, finally, generation of oxidative stress leading to ROS production (Santo et al., 2012; Zain et al., 2014), which has an antibacterial effect (Supplementary Figure S10).

#### Cytotoxicity assay of *P. nigrum* fruit extract and PN-CuNPs

CuNPs have numerous biomedical applications in medicine and drug delivery and have anti-angiogenic properties of cancer. PN-CuNPs and *P. nigrum* fruit extracts were subjected to cytotoxic effect investigation against the MCF-7 breast cancer cell line by MTT assay. PN-CuNPs exhibited cytotoxicity against cancer cells. At 200 μg/mL, PN-CuNPs showed cytotoxic activity of approximately 80% against the MCF-7 cell line (Supplementary Figure S11). The results shown in Supplementary Figure S11 indicated that PN-CuNPs caused a significant decrease in the viability of the MCF-7 cells when compared to the *P. nigrum* fruit extract (Chung et al., 2017; Lovika et al., 2021). The correlation graph between antioxidant and cytotoxic activity indicated that both assays are statistically linked with each other (Supplementary Figure S12).

The anticancer activity of CuNPs synthesized using a green process that involves medicinal plants was confirmed by previous studies (Jacob et al., 2012; Vivek et al., 2012; Suman et al., 2013). The study of Suman et al. (2013) clarified that the HeLa cell line was suppressed at high doses of *Morinda citrifolia* metallic NPs. *Piper longum* leaf extract NPs inhibited Hep-2 cell lines (Sankar et al., 2014). *Annona squamosa* leaf extract NPs suppressed MCF-7 cell lines (Greenwell and Rahman, 2015). The anticancer properties of CuNPs were influenced by the size, form, and surface coating of NPs. The size of CuNPs is the most influential of these parameters (Phaniendra et al., 2015). The photocatalytic and cytotoxic activity of nickel oxide nanoparticles and nickel nanosheets was evaluated using *Salvia hispanica* seed extract and tragacanth gum (Sabouri et al., 2020; Sabouri et al., 2021).

#### Proposed mechanism of cytotoxicity activity of PN-CuNPs

The significant anticancer potential of PN-CuNPs synthesized by *P. nigrum* fruit extracts against common MCF-7 breast cancer cell lines is linked to their antioxidant activities. Previous similar studies revealed that CuNPs and medicinal plants reduced tumors by suppressing free radicals (Greenwell and Rahman, 2015). Free radicals induced mutations in DNA and RNA, resulting in altered gene expression and, finally, leading to a proliferation of cancerous cells (Phaniendra et al., 2015). Elevated levels of free radicals in the various organs led to angiogenesis and tumorigenesis (Hielscher and Gerecht, 2015). The proposed mechanism of the cytotoxic activity of PN-CuNPs is depicted in Supplementary Figure S13. Medicinal plant-based PN-CuNPs inhibit cancer cell growth (Desai et al., 2008). In the current study, the viability of MCF-7 cells was reduced, suggesting anticancer activity of PN-CuNPs. Understanding the detailed mechanism requires further study.

#### Docking interactions

Glutathione reductase was used as a binding enzyme to virtually investigate the potential of the selected compounds as antioxidants. It is responsible for providing reduced glutathione to most cells and can control ROS.

**FIGURE 4:**
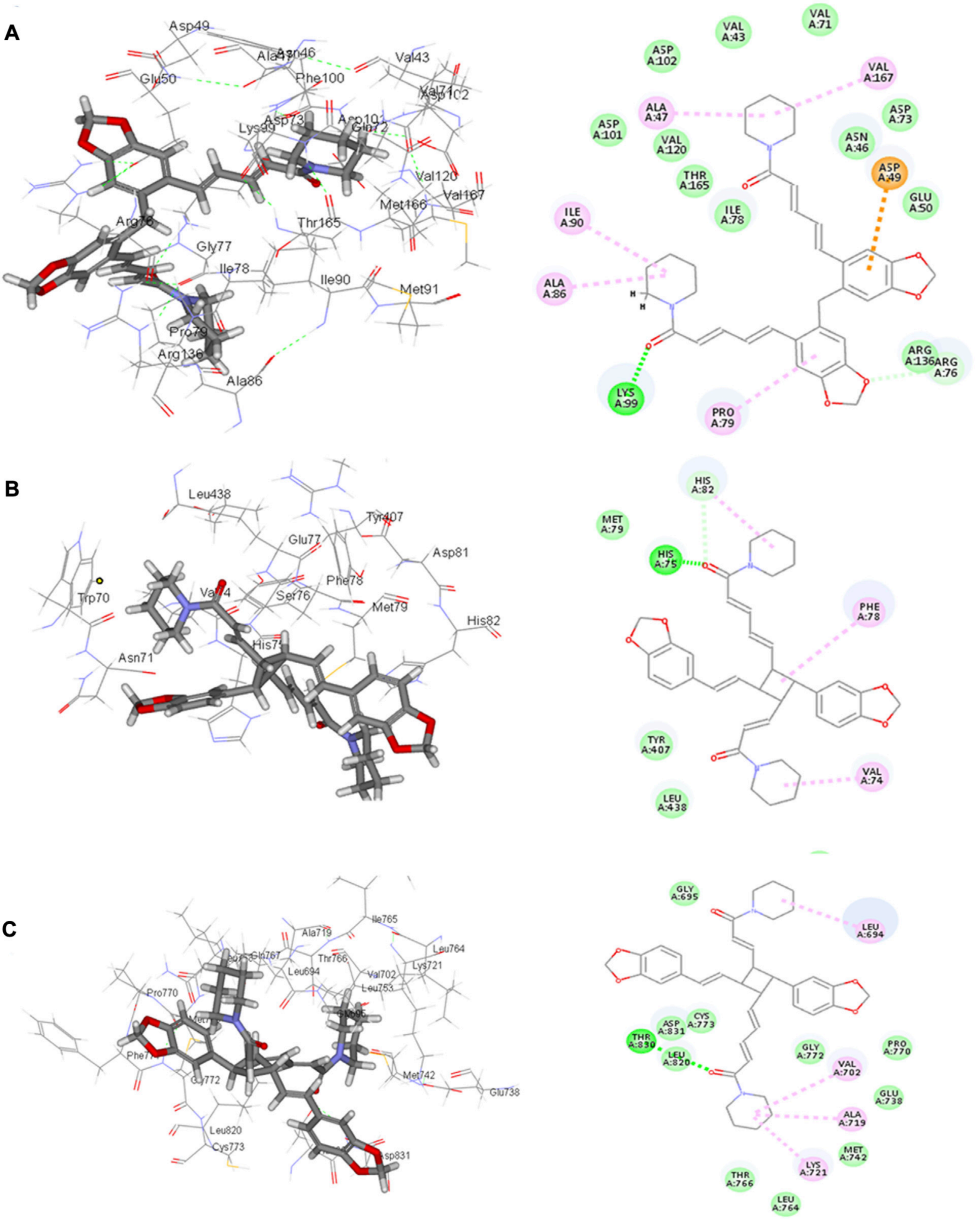
2D and 3D binding of the most potent compound 32 in the binding pockets of **(A)** glutathione reductase, **(B)** DNA gyrase topoisomerase II, and **(C)** EGFR tyrosine kinase.

Four compounds, namely, pipernigramide F 32, pipercyclobutanamide A 26, guineensine 10, and brachyamide A 40, reported the best binding free energy in the glutathione reductase enzyme as non-competitive inhibitors by fitting allosterically to the NADPH/FAD binding pocket. The scored ΔG values were 40.18, 41.83, 38.58, and 42.03 kcal/mol, respectively, while the ascorbic acid control scored 25.31 kcal/mol. The key amino acid residues involved in the noncovalent interactions were Asn71, His75, and His82, with both the native ligand HXP xanthene and ascorbic acid. Pipernigramide F 32 manifested seven firm interactions with the vital amino acid residues in the active site through its cyclobutane and aromatic rings together with the proton acceptors as the dioxole and carbonyl moieties detailed as a π–π -stacked bond with His75, a C-H bond with His82, π–alkyl bonds with His82, Trp70, and Phe82, a conventional H bond with Asn71, and a π–π T-shaped bond with Tyr407 (Figure 4A). Pipercyclobutanamide A 26 revealed eight noncovalent binding modes, which were π–sigma bonds with Phe78, three π–alkyl bonds with Phe78, Leu438, Val74, and Tyr407, C-H bonds with His75, π–donor H bonds with His82, and conventional H bonds with His75. Guineensine 10 showed several desirable interactions as five π–alkyl bonds with Tyr407, His82, Leu438, Val74, and Trp70, one π–π --stacked bond with Phe78, two conventional H bonds with Asn71 and His75, and one π–sigma bond with Phe78.

Brachyamide A 40 demonstrated π–π-stacked bonds with Phe78, C-H bonds with Glu77, His75, Tyr407, and His82, and alkyl bonds between the hydrophobic residues Leu438, Val74, Phe78, and the respective non-polar portions of the ligand. Additionally, Van der Waals forces were revealed with Tyr407.

The same interactions were revealed clearly using the Maestro module of the Schrodinger platform by applying the Glide XP docking protocol where the top 9 highest scoring compounds were 31 (pipernigramide E), 30 (pipernigramide D), 32 (pipernigramide F), 26 (pipercyclobutanamide A), 43 (pipwaqarine), 6 (piperchabamide C), 53 (kusunokinin), 41 (tricholein), and 19 (sylvamide). H bond interactions and π–π interactions were shown by Phe78, Phe87, Tyr85, His8, His75, and Tyr407 (Table 1).

**TABLE 1:** A list of the top 9 scoring compounds with their ∆G values and RMSD values in the binding sites of 1Kzn, 1Xan, and 1m17.

	cpd no.	1kzn (Kcal/mol)	RMSD	cpd no.	1Xan (Kcal/mol)	RMSD	cpd no.	1m17 (Kcal/mol)	RMSD
1	40	−5.196	2.9876	31	−5.419	1.9077	32	−5.652	1.5394
2	43	−5.024	3.1578	30	−5.535	4.6105	33	−5.134	1.2891
3	33	−4.725	2.8664	26	−4.253	0.9806	31	−5.762	3.4945
4	6	−4.97	1.7102	32	−4.705	1.5133	26	−4.417	1.1461
5	10	−4.925	2.8472	43	−4.543	3.0234	43	−4.823	3.4273
6	31	−5.45	3.2337	6	−4.368	1.5492	27	−4.531	1.7643
7	32	−4.483	1.6033	53	−5.222	1.5612	53	−7.12	2.3697
8	53	−4.193	0.8207	41	−4.062	2.4057	40	−4.46	2.2324
9	13	−4.58	2.7777	19	−4.13	2.4011	13	−4.465	3.1836

The importance of the *E. coli* DNA gyrase topoisomerase II (1Kzn) has become clear as it is involved in maintaining the chromosomal super helicity in the microbial transcription and replication processes. While the B subunit of the 1Kzn molecule encompasses the ATPase active site in the N-terminal domain, the C-domain interacts with the DNA to alter the super-helical form of the DNA substrate. Due to the rise in the number of resistant microbial strains, new inhibitors of the gyrase enzyme are desperately needed. Herein, the docking of the selected *P. nigrum* fruit components was performed in the ATP hydrolysis binding site, and amino acid residues with the most influence, as detailed and described by Gross et al., (2003), were identified. D73 and N46 are reported to synchronize with Mg^+2^ in the ATP binding site. The best binding bioactive compounds in the DNA gyrase active site are pipernigramide G 33, pipnoohine 25, pipercyclobutanamide A 26, and pipernigramide G 33. The amide group in compound 33 interacted through nine non-covalent bonds; a conventional H bond with Lys99; the aromatic and saturated rings exhibited four alkyl interactions with Ala86, Pro79, Val167, and Ala47; Van der Waals (vdW) forces between Arg136 and the dioxole ring. C-H bonds with Arg76 and between proton donors and Asp101 were recorded in the DNA gyrase pocket as well as a π–anion interaction with Asp49 (Figure 4B). The ΔG binding energy was –58.615 kcal/mol, which is superior to the positive control ampicillin (−49.407 kcal/mol) (Supplementary Table S1).

Pipnoohine 25 revealed a conventional H bond between Arg136 and the amide group. Alkyl interactions between the hydrophobic side chain, Ala86, and Pro79 were detected. Pipercyclobutanamide A 26 showed a conventional H bond with Gly77, and a π–anion interaction between Glu50, Arg76, and the aromatic ring. Pro79, Ile78, Ile90, Ala86, and Arg76 were involved in alkyl-type interactions with unsaturated and saturated rings. A π–π T-shaped bond was identified between Phe50 and the benzo dioxole ring, and a C-H bond was identified between Asp73, Asn46, and Asp49 and proton donors.

The core interacting residues in the standard ampicillin within 3 Å were a conventional H bond between the amine group and residue Asn46, attractive charges between Lys99 and the acidic anions, and vdW forces and alkyl interactions with Ile78 and Asp49. The aforementioned amino acids comparably interacted with the selected compounds, especially pipernigramide G 33, pipnoohine 25, and pipercyclobutanamide A 26, confirming their potential potency as antimicrobial agents.

Using the Xtra precision protocol of the Maestro module of the Schrodinger platform, similar polar and non-polar interactions were detected and verified with their comparative binding energies. Residues such as Arg136, Gly77, Thr165, and Arg76 represented core H bond interactions, while vdW interactions were noted with Asp49, Arg136, Gly77, Thr165, Asp49, Asn46, Ile90, Met166, and Val167. The best poses were selected based on the lowest binding energies, lowest RMSD values, and favorable interactions with the native ligand.

As far as EGFR tyrosine kinase (1m17) is concerned, the docking study was carried out in the same active site, the ATP binding site, as the native co-crystallized ligand erlotinib, a major tyrosine kinase inhibitor approved by the FDA (Sharifi-Rad et al., 2022). After careful analysis, the binding modes of pipernigramide G 33 and pipernigramide F 32 (Figure 4C) proved to be promiscuous and scored the lowest Gibbs free energy, −54.1347 and −62.1958 kcal/mol, respectively, with a minus sign indicating the quick auspicious fitting in the EGFR binding pocket, which is even better than erlotinib, whose free energy was −47.3483 kcal/mol (Supplementary Table S1). Within a measured distance of 2 Å, compound 33 showed a conventional H bond formation between the residues Lys721, Asp831, Thr830, and Thr766 and its amide carbonyl group. This was also evident between Met769 and the oxygen atom of the dioxole rings.

Four alkyl interactions between the aromatic moiety and four amino acid residues, Val702, Leu820, Ala719, and Leu694, stabilized the binding of the molecule over the hydrophobic bed. vdW forces between the piperidine protons and Pro770 added more to the non-polar favorable interactions; additionally, a π–donor hydrogen bond between Cys773 and the hydrophobic aromaticity and a C-H bond detected between Glu738 and the proton donors of the piperidine ring were identified.

The lowest ΔG value was reserved to pipernigramide F 32 (Figure 4C) with interaction forces as follows: a π–sulfur bond between Cys773 and the aromatic rings; conventional H bonds between the benzo dioxole ring and Lys721, Met769, Thr830, and Thr766; three C-H bonds with Pro770 and Glu738; alkyl interactions between the piperidine ring and Val702, Ala719, and Gly772; and a C-H bond noticed between the Gly695 and the dioxole ring. Moreover, Asp831 interacted through a C-H bond with the protons of the piperidine ring.

**FIGURE 5:**
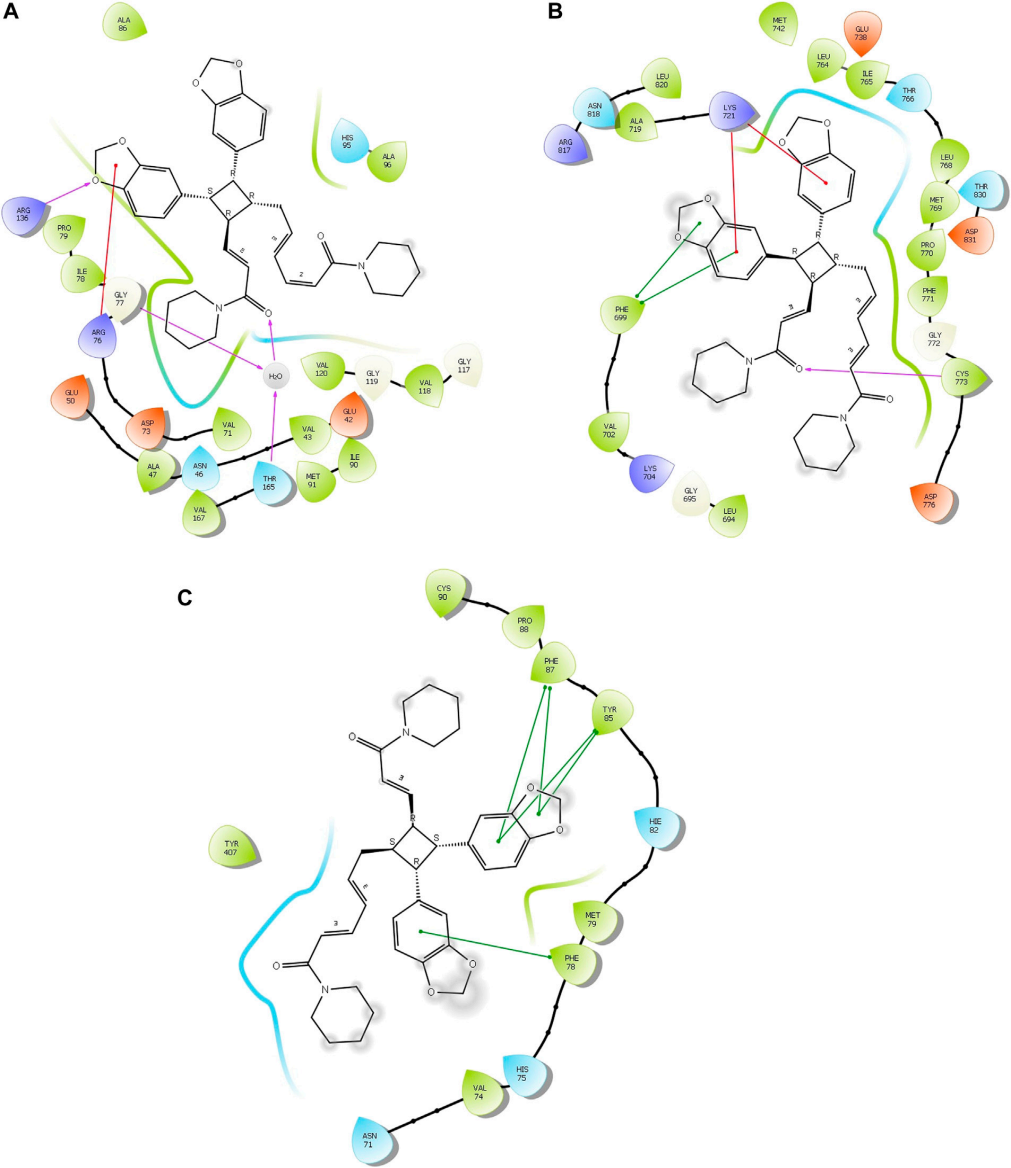
Compound 33 in targets **(A)** 1Kzn, **(B)** 1m17, and **(C)** 1Xan.

**FIGURE 6:**
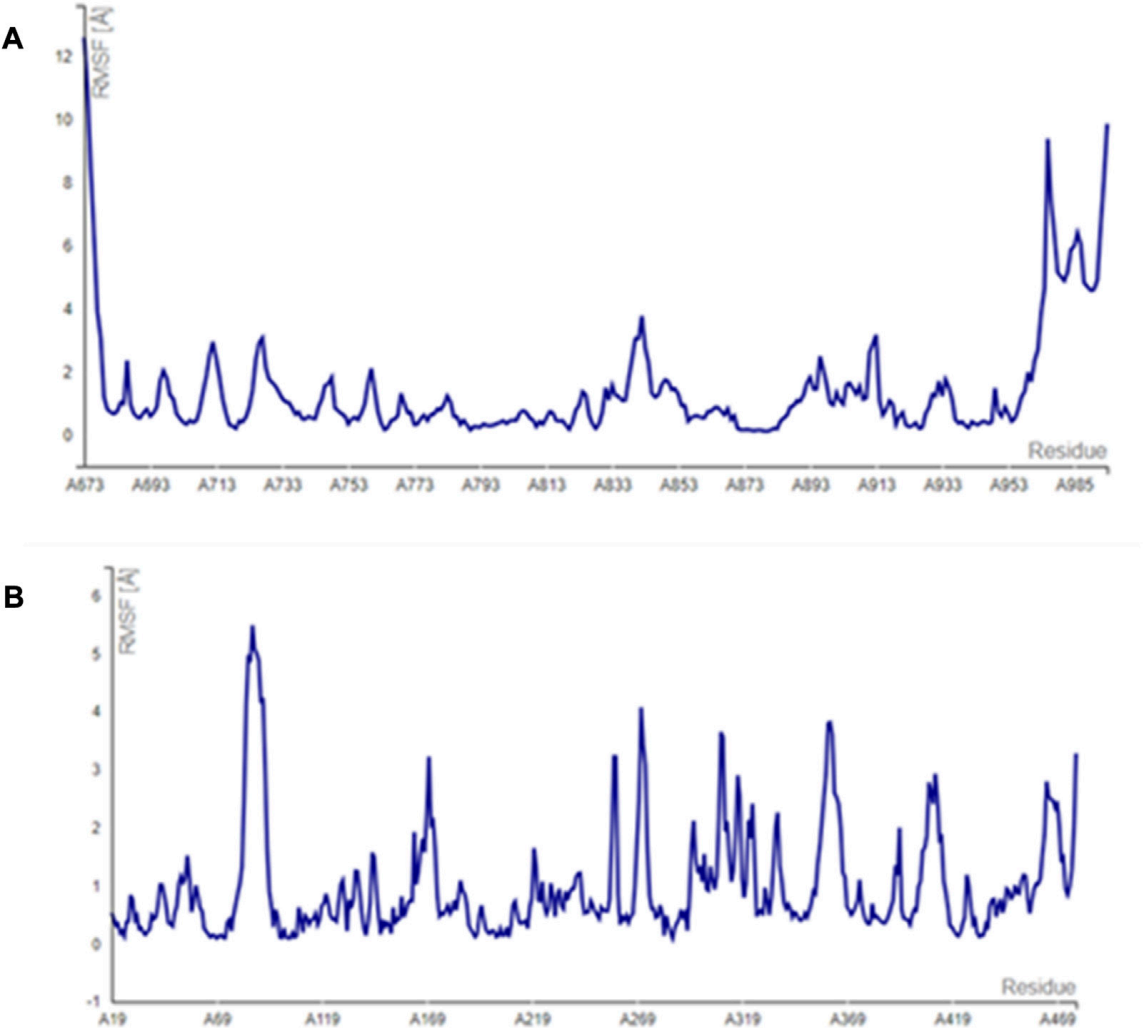
Simulation graphs revealing the stability of the protein targets **(A)** 1m17 and **(B)** 1Xan.

The main vital forces responsible for bioactivity were the number of different interactions, their types, bond distance measured, and strength as compared to the ligand erlotinib. Erlotinib’s good binding affinity comprised its unique interactions, namely, a salt bridge formation with Asp831, alkyl interactions with Lys721 and Met742, Leu820, and Val720, and an H bond with Cys773. vdW forces contributed to a firmer binding through Gly695, Glu738, and Leu694, all within a distance of less than 2 Å. Comparable interactions were identified in the bioactive compounds, particularly, pipernigramide F 32 and pipernigramide G 33, suggesting their promising potential as antitumor agents with tyrosine kinase inhibition activity (Figure 5). Protein stability was measured through flexibility studies manifesting 10 modules with RMSD values within the range of 2–4 Å, which suggested the stability of protein residues during interactions (Figure 6).

The vital factors that contributed to better binding forces and whose significance was most noted were the length of the molecule in terms of the number of carbons in the side chain; for instance, 26 was of firmer binding energy than pipernigramide F 32 owing to the only difference between them, which was the side chain length. Second, the presence of the piperidine ring improved fitting into the enzyme pocket as shown by comparing the ΔG value of brachyamide A 40 and guineensine 10. Even the unsaturation in a single bond contributed to a noticeable decrease in binding strength as is clear in the pairs of compounds 4/5, 16/17, and 13/14. Moreover, the size of the molecule and its orientation in the active site proved to be of considerable importance as is evident from the smallest three docked molecules, piperonal 24, 1-nitrosoimino-2,4,5-trimethoxybenzene 42, and 3′,4′-methylenedioxycinnamaldehyde 45, whose binding forces were the least compared to their congeners. It appeared that appropriate contact with the hydrophobic bed was essential for achieving the best possible binding interactions, which required either long chain molecule or a multicyclic structure; additionally, pyrrolidine was favored over the piperidine ring as observed from the two compounds, 1-piperettylpyrrolidine 36 and piperoleine 37 (Mafuné et al., 2002). The standard ascorbic acid proton acceptor groups showed conventional hydrogen bonds with His75, Asn71, and His82, which highlighted the core value of the number of proton acceptors in the binding ligand. It is worth mentioning that the best fitting compounds 32, 26, 10, and 40 manifested the same types of interactions with the amino acid residues. Similarly, the native ligand HXP xanthene revealed analogous types of interactions to the ascorbic acid control.

Binding interactions in the 1m17 were studied using Xtra precision protocol in the Maestro module of the Schrodinger platform, which depended on the intricately calculated “XP Pose Rank” property based on both the Glide score and the E model scores.

The key amino acids involved in interactions were Lys721, Asp831, Thr830, Met769, Cys773, and Pro770, confirming the poses obtained before with the C-Docker protocol (Table 1). The molecular modeling study was further validated using the Maestro module of the Schrodinger platform and indicated the top 9 compounds to be 40 (brachyamide A), 43 (pipwaqarine), 33 (pipernigramide G), 32 (pipernigramide F), 31 (pipernigramide E), 53 (kusunokinin), 6 (piperchabamide C), 10 (guineensine), and 26 (pipercyclobutanamide A) in the three binding pockets of the three target enzymes. Their binding free energy ranged between −7.12 and −4.13 kcal/mol (Tables 1, 2).

**TABLE 2:** Heatmap showing the free binding energy of selected pepper compounds in the binding pockets of target enzymes.

Compound	1Kzn	1m17	1Xan
39	−4.547	−5.183	−4.271
Piperlactam S	−4.845	−5.331	−5.367
Kusunokinin	−4.193	−7.12	−5.222
Cepharadione A	−3.821	−6.995	−4.525
51	−4.513	−5.715	−4.683
Piperolactam D	−5.13	−6.631	−5.109
Pipernigramide D	−1.721	−6.226	−5.535
Chavicine	−3.718	−5.531	−4.642
Trichostachine	−4.539	−4.946	−4.03
Pipernigramide E	−5.45	−5.762	−5.419
Piperine	−4.618	−4.624	−4.388
Piperettine	−4.051	−5.429	−4.748
Piperanine	−3.553	−6.188	−4.724
Piperlonguminine	−4.057	−3.651	−4.354
Isopiperine	−3.945	−4.561	−4.32
Isochavicine	−4.654	−6.642	−4.309
3′,4′-Methylenedioxycinnamaldehyde	−3.934	−5.001	−3.842
Piperonal	−4.36	−5.261	−3.091
(2E,4E,8E)-Piperamide-C9:3	−3.169	−5.679	−4.218
Piptigrine	−3.509	−4.581	−3.153
Pipernonaline	−4.339	−5.042	−3.869
Retrofractamide D	−3.854	−5.393	−3.852
Pipernigramide A	−3.063	−3.998	−3.599
Tricholein	−4.225	−4.55	−4.062
Pipernigramide F	−4.483	−5.652	−4.705
Pipercide	−4.58	−4.465	−2.92
Brachyamide A	−5.196	−4.46	−3.36
Pipwaqarine	−5.024	−4.823	−4.543
1-Piperettylpyrrolidine	−3.247	−4.722	−3.698
Pipercyclobutanamide A	−2.492	−4.417	−4.253
38	−4.373	−5.319	−3.84
Pipercallosine	−4.551	−4.448	−3.999
1-Nitrosoimino-2,4,5-trimethoxybenzene	−4.193	−4.253	−3.238
Piperolein B	−4.861	−5.582	−3.689
Retrofractamide C	−3.738	−4.64	−2.404
Paprazine	−4.543	−5.33	−4.945
Piperchabamide C	−4.97	−3.868	−4.368
Pipnoohine	−3.1	−2.839	−2.264
Piperchabamide D	−4.252	−5.138	−3.931
Sarmentine	−4.103	−3.261	−3.1
Pipernigramide G	−4.725	−5.134	−3.742
Pipernigramide D	−4.308	−5.092	−4.935
Pipernigramide B	−3.982	−4.531	−4.386
Dehydropipernonaline	−3.511	−4.797	−3.15
Stigmast-4-ene-3,6-dione	−3.385	−2.841	−3.209
Stigmastane-3,6-dione	−3.887	−4.491	−3.377
Dehydroretrofractamide C	−3.836	−4.321	−3.922
Guineensine	−4.925	−4.346	−3.819
Retrofractamide A	−3.92	−4.074	−3.865
Pipernigramide C	−3.92	−4.074	−3.865
Pipericine	−3.077	−3.04	−3.397
Pipernigrester A	−3.435	−3.781	−3.519
Sarmentosine	−6.475	−8.419	−4.955
Sylvamide	−5.126	−5.093	−4.13
Pellitorine	−2.932	−3.141	−2.187
2,4-Tetradecadienoic acid isobutyl amide	−2.909	−1.141	−1.721
2,4-Dodecadienamide, N-(2-methylpropyl)-, (2E,4E)	−2.897	−2.342	−1.55

**FIGURE 7:**
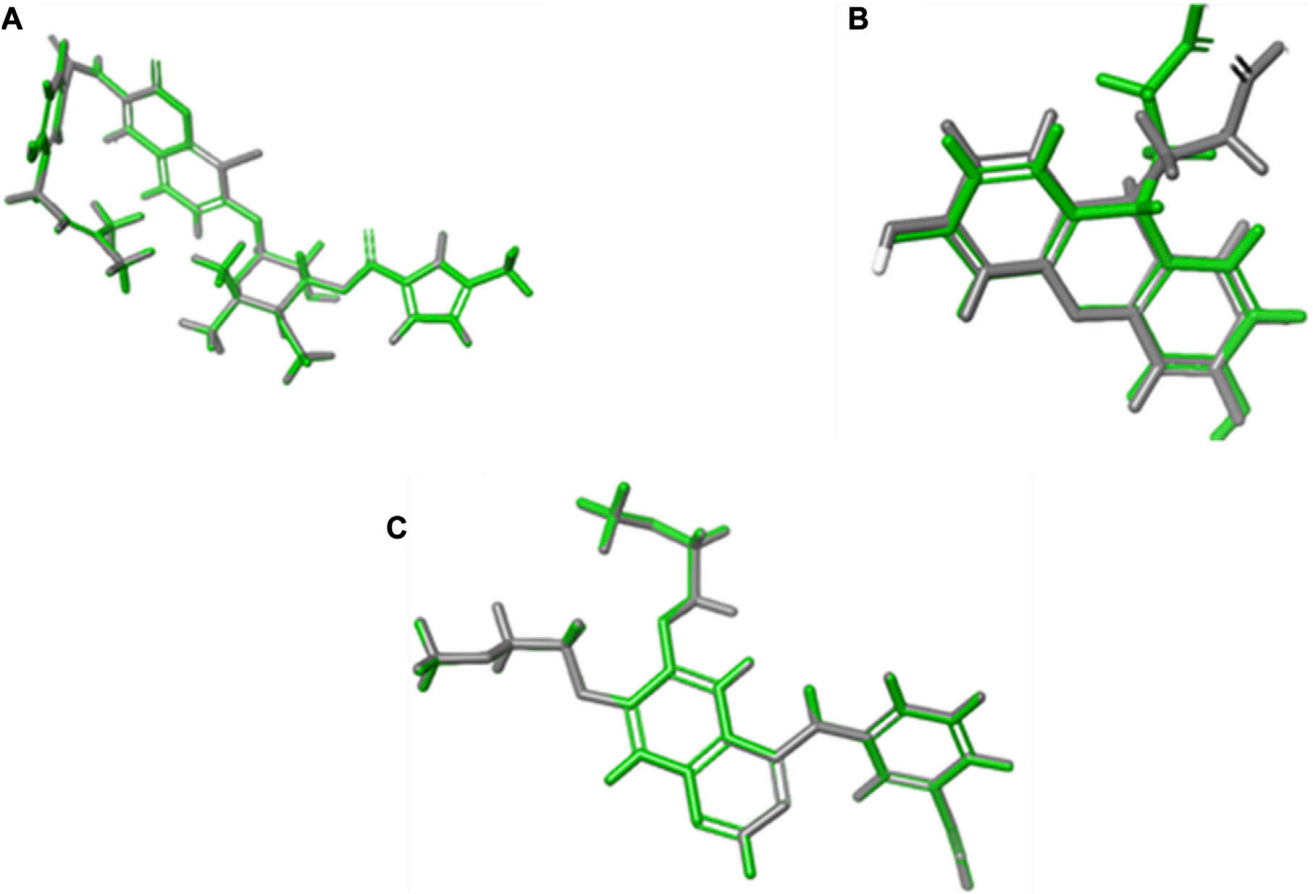
RMSD calculations through overlays between the native co-crystallized and redocked ligands. **(A)** 1Kzn, **(B)** 1Xan, and **(C)** 1m17 with the native ligand in green and docked ligand in gray.

Validation by redocking the co-crystallized ligands was performed in the three enzymes 1m17, 1Kzn, and 1Xan, revealing RMSD values of 0.0301, 0.0041, and 0.6626, respectively. RMSD values were calculated with limits indicating that accurate docking protocols were measured. The selection of compounds depended not only on the Gibbs free energy but also on the lowest RMSD values and favorable binding interactions, as explained in the following paragraphs (Figure 7).

The tremendous need to unveil new antibacterial molecules has become evident in the last 30 years when multiresistant organisms such as methicillin-resistant staphylococci (MRSA), vancomycin-resistant enterococci and staphylococci (VRSA), and erythromycin-resistant pneumonia began to spread widely. Natural products as a source of potent antimicrobial agents play a key role in the pursuit of new antibiotics. From the molecular modeling study and the proposed mechanisms, the antibacterial effect was regarded as largely based on the redox capacity of the PN-CuNPs (Cu^+1^ and Cu^+2^) through its interaction with SH groups, causing DNA damage and toxicity to bacteria. Furthermore, copper in high concentration can inhibit enzymes, block protein functional groups, and provide a source of hydroperoxide free radicals.

An antioxidant effect was evident through *in vitro* and *in silico* studies from the binding to the glutathione reductase human protein and the bacterial DNA gyrase. An anticancer effect was manifested by the favorable binding to the EGFR (PDB ID: 1m17) enzyme pocket manifested by H bonding and vdW force interactions through its Cys773 amino acid to a piperidyl amide group in most of the top-scoring compounds, which supported our proposed mechanism.

## ADMET and TOPKAT analysis

ADMET prediction of the selected alkamides indicated their pharmacodynamic, pharmacokinetic, and toxicity properties (Table 3). The cytochrome inhibition and hepatotoxicity profile were reported for only a few compounds. Penetration levels in the blood–brain barrier (BBB) were ranked from 0 to 4, indicating the very high penetration to the lowest levels. In the same way, absorption was expressed on the scale 0, 1, 2, and 3, indicating good, moderate, low, and very low absorption levels, respectively. The most potent compounds revealed a BBB penetration level of 4 with more than 90% binding to plasma proteins, indicating its promising drug likelihood (Table 3).

**TABLE 3:** ADMET properties of the tested alkamides in *Piper nigrum* fruits.

Compound	BBB level	Absorption level	Solubility level	Hepatotoxicity	CYP2D6	PPB level	ALogP98	PSA-2D
1	1	0	3	NT	NI	False	2.864	38.513
2	1	0	2	NT	NI	False	3.332	38.513
3	1	0	2	NT	NI	True	4.689	38.513
4	1	0	2	NT	NI	True	4.245	38.513
5	1	0	2	NT	NI	True	4.712	38.513
6	0	1	2	NT	NI	True	6.069	38.513
7	2	0	3	NT	NI	False	2.408	38.513
8	1	0	2	NT	NI	True	3.788	38.513
9	1	0	2	NT	NI	True	4.313	47.971
10	0	1	2	Tox	NI	True	6.138	47.971
11	0	0	2	NT	NI	True	4.87	30.111
12	1	0	2	NT	NI	False	3.823	38.513
13	0	0	2	Tox	I	True	5.226	47.971
14	0	0	2	Tox	I	True	5.67	47.971
15	1	0	3	NT	NI	False	3.958	30.111
16	1	0	2	NT	I	True	4.781	47.971
17	1	0	2	NT	I	True	4.758	47.971
18	3	0	4	NT	NI	False	2.333	71.741
19	2	0	2	Tox	I	True	2.461	55.814
20	1	0	2	NT	NI	True	3.773	44.091
21	3	0	2	Tox	I	True	2.326	68.786
22	2	0	2	Tox	I	True	2.639	70.814
23	3	0	2	Tox	I	True	2.332	71.031
24	1	0	2	NT	I	True	4.758	47.971
25	2	0	3	NT	NI	True	1.357	35.16
26	4	3	2	NT	NI	True	8.007	30.111
27	1	0	2	Tox	NI	True	4.847	77.027
28	0	0	2	NT	NI	True	5.625	38.513
29	2	0	4	NT	NI	False	1.23	37.954
30	3	0	3	Tox	NI	True	2.91	80.672
31	4	1	2	NT	NI	True	5.982	77.027
32	4	1	2	NT	NI	True	5.993	77.027
33	4	1	2	NT	NI	True	5.874	77.027
34	1	0	2	Tox	NI	True	4.45	47.971
35	2	0	3	NT	NI	True	2.933	47.971
36	1	0	2	NT	NI	False	3.332	38.513
37	1	0	3	NT	NI	False	2.876	38.513
38	1	0	2	NT	NI	True	3.788	38.513
39	2	0	3	NT	NI	False	2.397	38.513
40	0	0	2	NT	NI	True	5.613	38.513
41	1	0	2	NT	NI	True	4.256	38.513
42	3	0	3	NT	NI	False	1.609	66.737
43	4	1	2	Tox	NI	True	6.527	47.971
44	2	0	3	Tox	NI	True	2.926	71.741
45	2	0	3	Tox	NI	True	2.814	71.741
46	2	0	3	Tox	NI	True	2.814	71.741
47	2	0	3	NT	NI	True	1.717	35.16
48	4	3	0	NT	NI	True	7.318	34.601
49	4	2	1	NT	NI	True	6.936	34.601
50	0	0	2	NT	NI	True	5.782	30.111
51	4	3	5	NT	NI	False	−2.338	144.872
53	2	0	3	NT	NI	True	2.317	55.814
54	1	0	2	Tox	NI	True	3.77	61.951
55	2	0	2	Tox	I	True	2.394	59.328
56	4	3	2	NT	NI	True	7.607	30.111
57	1	0	2	NT	NI	True	3.309	38.513
58	1	0	3	NT	NI	False	2.864	38.513
59	2	0	3	Tox	I	True	2.532	62.036

**FIGURE 8:**
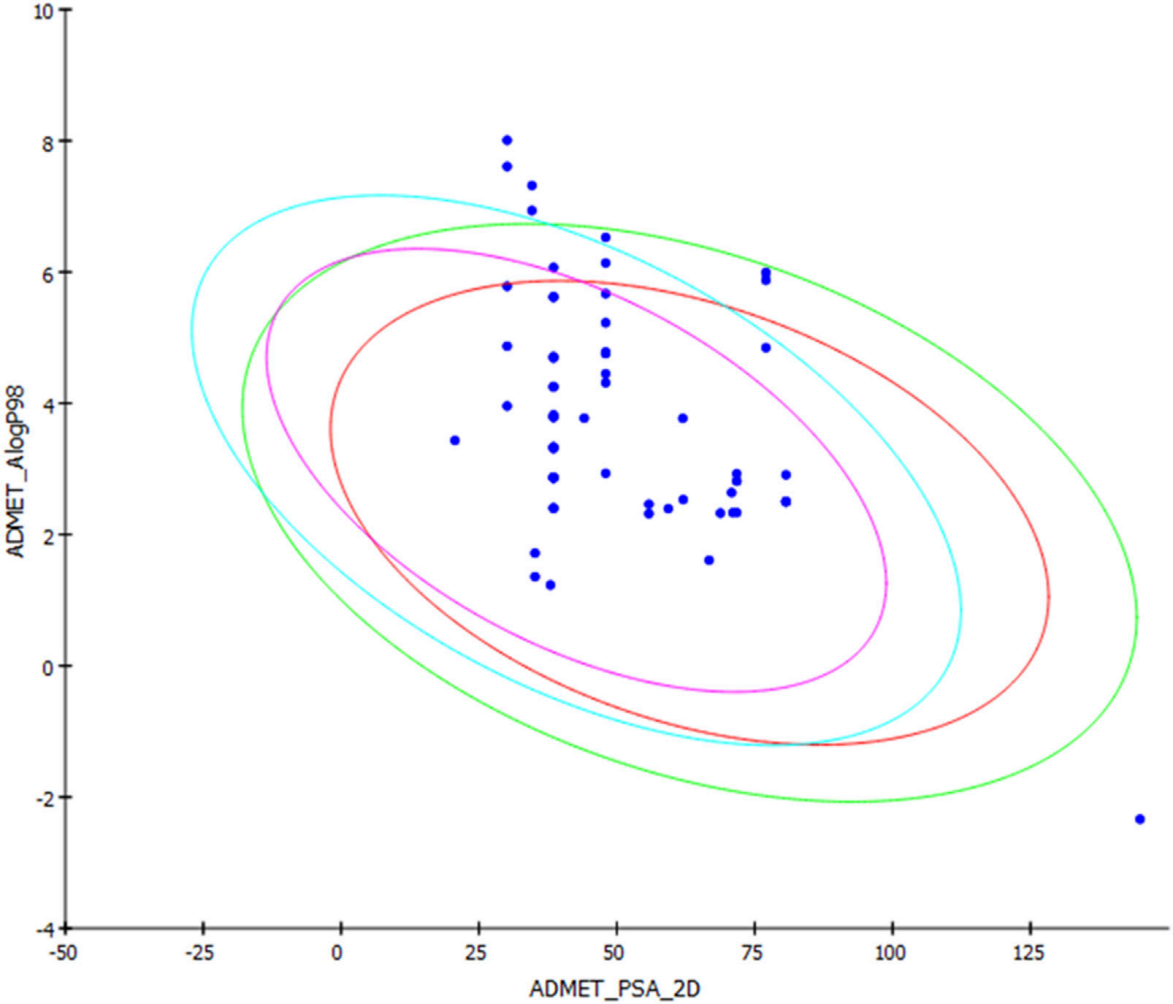
ADMET plot for chosen alkamides from *Piper nigrum* fruits, showing the blood–brain barrier (BBB) 99% and 95% confidence limit ellipses and the human intestinal absorption models in ADMET_AlogP98.

Pipernigramide E 31, F 32, and G 33 demonstrated moderate absorption, yet pipercyclobutanamide A 26 manifested a very low absorption score. The solubility score of 2 indicated low solubility for the compounds pipernigramide E 31, F 32, and G 33 and pipercyclobutanamide A 26, which possibly require a suitable formulation to assist delivery to the active site. Most of the alkamides tested revealed moderate intestinal absorption characteristics, as evidenced by the ADMET plot in Figure 8, where they were in the 99% absorption ellipse, with only compounds 52–54 located outside the 99% absorption range. BBB penetration with values of 0 and 1 was either very high penetration or high penetration, except for compounds 35–39, which fell outside the 99% confidence ellipse of BBB absorption (Table 3; Figure 8). Regarding plasma protein binding, most of the compounds showed more than 90% binding except for 1, 2, 7, 12, 15, 18, 29, 36, 37, 39, 42, 51, and 58. The solubility levels of the chosen compounds were of values 2 or 3, indicating low to moderate solubility, except for compounds 48 and 49, which were identified as being insoluble and of very low solubility levels. A few compounds manifested binding to CYP2D6, such as 13, 14, 16, 17, 19, 21–24, and 55.

The TOPKAT analysis revealed that all the tested compounds were non-mutagenic and non-carcinogenic against male and female rats FDA except for 18, 20–22, and 54 (Table 4). The LD_50_ measured orally in rats ranged between 0.11 and 4.81 g/kg body wt., whereas the lowest observed adverse effect level (LOAEL) values ranged between 0.296 and 0.0002 g/kg body wt. Ocular irritancy and skin irritancy were mild for most of the compounds.

**TABLE 4:** TOPKAT prediction of the tested alkamides from *Piper nigrum* fruits.

Compound	Ames prediction	Rat oral LD_50_	Rat chronic LOAEL	Skin irritancy	Ocular irritancy	Rat male FDA	Rat female FDA
1	Non-mutagen	0.699537	0.00656405	Mild	Mild	Non-carcinogen	Non-carcinogen
2	Non-mutagen	0.773324	0.00559316	Mild	Mild	Non-carcinogen	Non-carcinogen
3	Non-mutagen	1.07948	0.00295513	Severe	None	Non-carcinogen	Non-carcinogen
4	Non-mutagen	0.960009	0.00301254	Mild	Mild	Non-carcinogen	Non-carcinogen
5	Non-mutagen	1.63813	0.00248574	Severe	None	Non-carcinogen	Non-carcinogen
6	Non-mutagen	1.26178	0.00255341	Mild	None	Non-carcinogen	Non-carcinogen
7	Non-mutagen	0.831267	0.0163319	Mild	Mild	Non-carcinogen	Non-carcinogen
8	Non-mutagen	1.15027	0.00755798	Mild	Mild	Non-carcinogen	Non-carcinogen
9	Non-mutagen	1.99221	0.00801701	Mild	None	Non-carcinogen	Non-carcinogen
10	Non-mutagen	2.63527	0.011869	Mild	None	Non-carcinogen	Non-carcinogen
11	Non-mutagen	2.69314	0.0546533	Mild	None	Non-carcinogen	Non-carcinogen
12	Non-mutagen	1.47311	0.00205941	Severe	Severe	Non-carcinogen	Non-carcinogen
12	Non-mutagen	2.22155	0.0115162	Mild	None	Non-carcinogen	Non-carcinogen
13	Non-mutagen	2.4754	0.0239389	Mild	None	Non-carcinogen	Non-carcinogen
14	Non-mutagen	2.26097	0.0337304	Mild	None	Non-carcinogen	Single-carcinogen
15	Non-mutagen	2.24329	0.0163692	Mild	None	Non-carcinogen	Non-carcinogen
16	Non-mutagen	2.07479	0.0230936	Mild	None	Non-carcinogen	Non-carcinogen
17	Non-mutagen	2.12355	0.182032	Mild	None	Non-carcinogen	Non-carcinogen
18	Mutagen	0.871606	0.040755	None	Mild	Multi-carcinogen	Non-carcinogen
19	Non-mutagen	3.77773	0.00916935	Severe	None	Non-carcinogen	Non-carcinogen
20	Mutagen	2.20834	0.296757	None	Mild	Multi-carcinogen	Non-carcinogen
21	Mutagen	0.58896	0.178629	None	Mild	Single-carcinogen	Non-carcinogen
22	Mutagen	0.739978	0.207833	None	Mild	Single-carcinogen	Single-carcinogen
23	Non-mutagen	2.68972	0.0248511	Mild	None	Non-carcinogen	Non-carcinogen
24	Non-mutagen	0.720878	0.0463523	Severe	Severe	Non-carcinogen	Non-carcinogen
25	Non-mutagen	3.52932	0.045389	Mild	None	Non-carcinogen	Single-carcinogen
26	Non-mutagen	0.134026	0.000322202	Mild	Mild	Non-carcinogen	Non-carcinogen
27	Non-mutagen	1.85205	0.0025682	Severe	None	Non-carcinogen	Non-carcinogen
28	Non-mutagen	1.5891	0.0311345	Mild	Severe	Non-carcinogen	Single-carcinogen
29	Non-mutagen	5.76115	0.0157735	None	Severe	Non-carcinogen	Non-carcinogen
30	Non-mutagen	1.32768	0.105859	None	Severe	Non-carcinogen	Non-carcinogen
31	Non-mutagen	0.147778	0.000209789	Mild	Mild	Non-carcinogen	Non-carcinogen
32	Non-mutagen	0.442598	0.000262114	Mild	Severe	Non-carcinogen	Non-carcinogen
33	Non-mutagen	2.63857	0.00543712	Mild	None	Non-carcinogen	Non-carcinogen
34	Non-mutagen	1.54443	0.00765291	Mild	Severe	Non-carcinogen	Non-carcinogen
35	Non-mutagen	0.773324	0.00559316	Mild	Mild	Non-carcinogen	Non-carcinogen
36	Non-mutagen	0.922921	0.0139764	Mild	Mild	Non-carcinogen	Non-carcinogen
37	Non-mutagen	1.15027	0.00755798	Mild	Mild	Non-carcinogen	Non-carcinogen
38	Non-mutagen	0.257543	0.00739208	Mild	Mild	Non-carcinogen	Non-carcinogen
39	Non-mutagen	1.37968	0.00425613	Mild	None	Non-carcinogen	Non-carcinogen
40	Non-mutagen	1.78122	0.00412023	Severe	None	Non-carcinogen	Non-carcinogen
41	Non-mutagen	0.11136	0.00362488	None	Mild	Multi-carcinogen	Multi-carcinogen
42	Non-mutagen	2.62323	0.00647272	Mild	None	Non-carcinogen	Non-carcinogen
43	Non-mutagen	2.67955	0.025341	None	Severe	Non-carcinogen	Non-carcinogen
44	Non-mutagen	1.75339	0.154136	None	Severe	Non-carcinogen	Non-carcinogen
45	Non-mutagen	1.75339	0.154136	None	Severe	Non-carcinogen	Non-carcinogen
46	Non-mutagen	0.743252	0.0132063	Severe	Mild	Multi-carcinogen	Non-carcinogen
47	Non-mutagen	2.7863	0.059828	Severe	Mild	Non-carcinogen	Single-carcinogen
48	Non-mutagen	2.89133	0.0793748	Severe	Mild	Non-carcinogen	Single-carcinogen
49	Non-mutagen	3.12925	0.0580334	Mild	None	Non-carcinogen	Single-carcinogen
50	Non-mutagen	3.36806	0.0179123	Mild	Severe	Non-carcinogen	Non-carcinogen
51	Non-mutagen	1.82564	0.0184801	Mild	None	Non-carcinogen	Single-carcinogen
53	Non-mutagen	3.35849	0.127313	Severe	Mild	Non-carcinogen	Non-carcinogen
54	Mutagen	1.01416	0.22397	None	Mild	Multi-carcinogen	Non-carcinogen
55	Non-mutagen	4.81789	0.0635814	Mild	None	Non-carcinogen	Single-carcinogen
56	Non-mutagen	1.04811	0.00760525	Severe	Severe	Non-carcinogen	Non-carcinogen
57	Non-mutagen	0.699537	0.00656405	Mild	Mild	Non-carcinogen	Non-carcinogen
58	Non-mutagen	0.699537	0.00656405	Mild	Mild	Non-carcinogen	Non-carcinogen
59	Non-mutagen	0.699537	0.00656405	Mild	Mild	Non-carcinogen	Non-carcinogen

The antibiotic resistance problem has exceeded expectations with serious health decline and death rates [79]. Without significant intervention, more than 10 million people are estimated to die because of a lack of effective antibiotics against resistant microbial species. The use of adequately designed CuNPs in the medical field is a promising strategy to tackle multiresistant pathogens, especially if the use is supported with computational approaches to verify their molecular efficacy [80, 81].

## Conclusion

The green synthesis of CuNPs using an aqueous extract of *P. nigrum* fruit extract was confirmed by UV-vis absorption, EDX analysis, FT-IR spectrometry, and XRD diffraction pattern. The particle size of the synthesized PN-CuNPs was 30–32 nm with a zeta potential of approximately −50 mV. This study demonstrated that both *P. nigrum* fruit extract and synthesized PN-CuNPs displayed considerable antioxidant, antibacterial, and antitumor activities. Virtual screening techniques were employed on more than 55 alkamide/alkaloid compounds using different molecular modeling software: Discovery Studio 4.5 software and the validated C-Docker protocol and Maestro Schrodinger platforms. This unveiled the promising free binding energies of most of the compounds, which were superior compared to their native ligands. Careful analysis of docking results manifested key amino acid interactions and binding forces as well as vital factors involved in structure binding relationships. An ADMET/TOPKAT study indicated that the most potent compounds were of low toxicity and favorable pharmacodynamic and pharmacokinetic profiles.

## Data Availability

The datasets presented in this study can be found in online repositories. The names of the repository/repositories and accession number(s) can be found in the article/Supplementary Material.
