# Supplementary material for: Green synthesis of Piper nigrum copper-based nanoparticles: in silico study and ADMET analysis to assess their antioxidant, antibacterial, and cytotoxic effects
Source: Front Chem. 2023 Sep 5;11:1218588. doi: 10.3389/fchem.2023.1218588 (PMC10509375; doi:10.3389/fchem.2023.1218588)
Supplement: Supplementary file 1 [file DataSheet2.docx]

**Supplementary Material**

**Green Synthesis of *Piper nigrum* Copper-based Nanoparticles: *In-Silico* Study and ADMET Analysis to Assess Their Antioxidant, Antibacterial, and Cytotoxic Effects**

**Modumudi Kiranmayee ^a^, Nambi Rajesh^a^, M. Vidya Vani^a^, Habeeb Khadri^b^, Arifullah Mohammed^c^, Suresh V. Chinni^d,e^, Gobinath Ramachawolran^f*^, Khateef Riazunnisa^a*^, Ashaimaa Y. Moussa^g*^**

^a^Department Biotechnology and Bioinformatics, Yogi Vemana University Kadapa, Andhra Pradesh, India-516005

^b^Department of Medical Laboratories, College of Applied Medical Sciences, Qassim University, Qassim 51452, Kingdom of Saudi Arabia.

^c^Department of Agriculture Science, Faculty of Agro-Based Industry, Universiti Malaysia Kelantan,17600Jeli, Kelantan, Malaysia.

^d^Department of Biochemistry, Faculty of Medicine, Bioscience, and Nursing, MAHSA University, 42610 Jenjarom, Selangor, Malaysia

^e^Department of Periodontics, Saveetha Dental College and Hospitals, Saveetha Institute of Medical and Technical Sciences, Chennai, India

^f^Department of Foundation, RCSI & UCD Malaysia Campus, No 4, Jalan Sepoy Lines, 10450 Georgetown, Pulau Pinang, Malaysia.

^g^Department of Pharmacognosy, Faculty of Pharmacy, Ain shams University, Cairo, Egypt, 11566

*****Corresponding Authors1: Ashaimaa Y. Moussa, [Ashaimaa_yehia@pharma.asu.edu.eg](mailto:Ashaimaa_yehia@pharma.asu.edu.eg) (AYM)

*Corresponding Author 2: Khateef Riazunnisa, [khateefriaz@gmail.com](mailto:khateefriaz@gmail.com) (KR)

* Corresponding Author 3: Gobinath Ramachawolran, [r.gobinath@rcsiucd.edu.my](mailto:r.gobinath@rcsiucd.edu.my)

| Compounds | XAN ΔG(Kcal/mol) | Interactions | KZN ΔG(Kcal/mol) | Interactions | 1m17ΔG(Kcal/mol) | Interactions |
| --- | --- | --- | --- | --- | --- | --- |
| 1 | 25.0137 | H-bond: Asn71, C-H bond: His82, Pipi-stacked: Phe78, Alkyl: His75, Val74 | 35.6456 | Pi-sigma Asn46, alkyl: Ile78, Val167, Pro79, Ala86, Ile90 | 36.9358 | H-bonds: Lys721, alkyl: Ala719, Leu764, Met742, Leu820, Val702 |
| 2 | 27.2738 | H-bond: Asn71, C-H bond: His82, alkyl; His76, Val74, Pi-pi stacked: Phe78 | 37.6182 | H-bonds: Gly77, Lys99 alkyl: Pro79, Ala86, Ile78 | 34.0067 | H-bonds: Asp831, Lys721, VDW: Asp831, Pro770, alkyl: Val702, Leu820, Ala719, Leu694 |
| 3 | FD |  | 40.8963 | H bonds: Lys99, alkyl: Pro79, Ile90, Ile78 | 38.9784 | H-bonds: Lys721, alkyl: Ala719, Leu694, Val702, Leu820 |
| 4 | 29.0223 | H-bond: His75, C-H bond: His82, Pi-pi stacked: Phe78, alkyl: Val74, Leu438, Thr407 | 41.8572 | Pi-cation: Lys99, alkyl: Pro99, VDV: Asn46 | 40.7249 | H-bonds: Lys721, C-Hbonds: Glu738, alkyl: Met742, Leu764, Val702, Ala719, Leu820, Cys773 |
| 5 | 32.4417 | C-H bondLThr407, His82, Pipi stacked: Phe78, Alkyl: Val74, His75 | 43.7449 | H bond: Asn46, alkyl: Ala47, Val167, Ile90 | 39.9845 | H-bond: Lys721, C-H bond: Asp831, Pro770alkyl: leu820, Val702, Ala719, Leu694 |
| 6 | 33.8603 | PiPi-stacked: Phe78, Alkyl: Phe78, Val74, His82 | 46.3342 | Pi-cation: Arg136, alkyl: Ala47 ,Val167, Ile90, Ile78, Val48 | 47.1325 | Pi-sulphur bond: Cys773, C-H bond: Asp831, alkyl: Leu820, leu694, Lys721, Val702 |
| 7 | 25.6997 | H bond: Asn71, C-H bond: His82, Thr407, Pipi-stacked: Phe78, Alkyl: His82 | 36.8669 | H bond: Arg136, alkyl: Val120, Val43, Ile78, Pi-stacked: Asn46 | 35.085 | alkyl: Leu820, leu694, Lys721, Val702, Cys773, Ala719, Met742, |
| 8 | 31.7709 | C-H bond: Phe78, Pi-pi-T shaped: Trp70, alkyl: His82, His75 | 38.8255 | H-bonds: Arg136, alkyl: Lys99, Pro90, Ile78 | 38.1224 | H-bond: Lys721, C-H bond: Glu738, alkyl: Leu820, Val702, Cys773, Ala719, Met742, |
| 9 | 29.3638 | C-H bond: His75, alkyl: Val74, Phe78, Trp70 | 40.3533 | Hbond: Asp102, alkyl: Ile78, Val167, Pro79, Ala86, Ile90 | 38.8898 | H-bond: Lys721, C-H bond: Glu738, alkyl: Leu820, Val702, Ala719, Met742, Leu769 |
| 10 | 38.5811 | H-bond: Asn71, Pi-pi stacked: His82, alkyl: Val74, Trp70, Phe78 | 48.2001 | Hbond: Arg136, alkyl: Pro90, Ile78, Val167, Val43 | 42.6051 | alkyl: Leu820, leu694, Lys721, Val702, Cys773, Ala719 |
| 11 | 26.7517 | H-bond: Asn71, Alkyl: Trp70, His75, Phe78, His82, Tyr407 | 34.2642 | C-H bond: Pro79, alkyl: Ile78, Val167, Ala86, Ile90 | 35.0505 | H-bond: Lys721, Asp831, alkyl: Cys773, Leu694, unfavourable: Thr830 |
| 12 | 31.012 | Asn71, Pipi-T shaped His82, C-H bond: Tyr407, alkyl: Val74, His75 | 40.8819 | C-H bond: Asn46, alkyl: Val167, Ala86, Ile90, Lys99, Pro79 | 37.4807 | H-bond: Lys721, Pi-sigma: Gly772, alkyl: Leu820, Cys773, Val702 |
| 13 | 33.5067 | H bond: Asn71, alkyl: His82, Tyr407, Val74, His75, Phe78, Leu438 | 47.0846 | C-H bond: Asp73, alkyl: Val167, Ala47, Ile90, val43, Val120, Ala86 | 40.3589 | H-bond: Lys721, Asp831, Met769, alkyl: Leu820, Leu694, Val702, Ala719 |
| 14 | 35.0562 | Pipi-stacked: Phe78, Alkyl: Tyr407, Val74, His75, Phe78, Leu438 | 44.8954 | H-bond: Asn46, alkyl: Val167, Ala47, Ile90, Val120, Ala86, Ile78 | 43.7208 | H-bond: Met769, Pi-sigma, Leu694, alkyl: Lys721, Leu820, Leu694, Ala719 |
| 15 | 25.3241 | H bond: Asn71, alkyl: His82, Tyr407, His75, Phe78 | 35.4024 | H-bond: Arg136, alkyl: Ile78, Val120 | 29.6149 | H-bond: Thr766, alkyl: Lys721, Leu820, Leu694, Val702 |
| 16 | 32.2409 | H bond: Asn71, Pipi-Tshaped: Tyr407, Pipi-stacked: Phe78, alkyl: Leu438, His75 | 43.1292 | H-bond: Lys99, Pi-stacked: Asn46, alkyl: Ile90, Val120, Ala86, Ile78, Pro79, Val43 | 40.7077 | H-bond: Asp831, C-H bond: Glu738, alkyl: Lys721, Leu820, Leu694, Val702, Ala719, Met769 |
| 17 | 30.0778 | H bond: Asn71, Pipi-stacked: His82, alkyl: His75, Phe78 | 40.9804 | H-bond: Arg136, alkyl: Ile90, Val167, Ile78, Pro79, Val43, Lys99 | 39.2926 | H-bond: Lys721, C-H bond: Asp831, Alkyl: Leu820, Leu694, Val702, Ala719, Leu768 |
| 18 | FD |  | FD |  | FD |  |
| 19 | 30.1387 | C-H bond: His75, Alkyl: Phe78 | 38.1263 | H-bond: Glu50, alkyl: Val120, Val43, Pro79 | 35.4922 | H-bond: Thr766, Thr830, C-H bond: Gly772, Alkyl:Leu694, Val702, Lys721 |
| 20 | 24.0127 | Pipi-stacked: Phe78, Pipi-T shaped: His75, alkyl: Val74 | 38.0161 | amide Pi-stacked: Gly77, alkyl: Ile90, Ile78, Pro79, Lys99, Arg76 | 35.6556 | H-bond: Lys721, Pi-sigma: Val702, Pi-anion: Asp831, alkyl: Leu820, Leu694, Cys773, Ala719, Leu768 |
| 21 | 29.147 | Alkyl: His82, Val74, Phe78 | 36.6837 | H-bond: Arg136, amide Pi-stacked: Asn46, C-H bond: Arg76, Asp101, Alkyl: Val120, Val167, Ile78, Val43 | 35.3723 | H-bond: Cys773, Pi-cation: Lys721, alkyl: Leu820, leu694, Val702, Met742 |
| 22 | 23.7547 | Pipi-stacked: Phe78, Pipi-T shaped: His75, alkyl: Val74, Tyr407, C-H bond: His82 | 38.3938 | C-H bond: Lys99, Amide Pi-stacked: Asn46, Alkyl: Ile78, Ile90, Pro79 | 33.0099 | H-bond: Thr766, Alkyl: Leu820, leu694, Cys773, Ala719, Leu768, Leu768, Val702 |
| 23 | 29.7275 | H-bond: Asn71, Alkyl: Leu438, His82, Tyr407 | 39.7317 | H-bonds: Arg136, Pi-sigma: Lys99, alkyl: Pro79, Ile78, C-H bond: Arg76 | 35.5897 | Alkyl: Leu820, leu694, Met742, Ala719, Val702 |
| 24 | 15.2367 | H bond: His75, Pipi-stacked Phe78, C-H bond: His82, | 20.6258 | H-bond: Arg136, C-H bond: Glu60, Alkyl: Lys99, Pro79, Ile90 | 20.4246 | H-bond: Met742, C-H bond: Asp831, Alkyl: Leu820, Val702 |
| 25 | 39.5821 | Alkyl: His82, Val74, Phe78, His75, Tyr407, Leu438 | **50.4095** | **H-bond: Arg136, C-H bond: Glu60, Alkyl: Ile90, Val167, Ile78, Val43, Lys99** | 45.0558 | Alkyl: Leu820, Lys721, Ala719, Val702 |
| 26 | **41.8344** | **C-H bond: His75, His82, Tyr407, alkyl: Met79, Leu438, Trp70** | **50.4892** | **Pi-anion: Glu50, Pi-sigma: Pro79, alkyl: Arg76, Ile78, Ile90, Lys99, Ala86** | **51.0444** | Pi-sulphur: Cys773, Pi-sigma: Gly772, C-H bond: Phe771, Asp831, Alkyl: Lys721, Val702 |
| 27 | 33.4747 | H-bond: His75, C-H bond: Tyr407, His82, Pipi-T shaped: Phe78, alkyl: Val74, Met79 | 47.1543 | C-H bond: Ala86, alkyl: Ala47, Val167, Pro79, Ile90, | 46.3743 | H-bond: Lys721, Alkyl: Leu820, Leu694, Val702 |
| 28 | 23.0384 | H-bond: Asn71, His75, C-H bond: His82, Alkyl: Phe78 | 26.7425 | C-H bond: Glu50, Asp73 | 25.6469 | C-H bond: Asp831, Alkyl: Leu820, Val702,  Ala719 |
| 29 | FD |  | FD |  | FD |  |
| 30 | 30.8444 | Pipi-T shaped: Tyr407, Pipi-stacked: Phe78, C-H bond: His82, Alkyl: Val74 | 40.7395 | H-bond: Arg136, Asp73, Pi-sigma, Asn46, alkyl: Pro79, Ile78 | 41.6041 | H bond: Met769, Glu738, C-H bond: Pro770, Pi-cation: Lys721 |
| 31 | 36.9569 | H bond: Asn71, Alkyl: His75, Phe78 | 58.1283 | H-bond: Gly77, Asn46, Alkyl: Ala86, Pro79, Ile78, Ile90, Lys99, | **54.0315** | H-bond: Lys721, C-H bond: Pro770, Leu694, Glu738, amide Pi-stacked: Gly695, alkyl: Cys773, Leu820, Val702, Met742 |
| 32 | 40.1896 | Pipi-stacked: His75, C-H bond: His82, alkyl: Trp70, Phe78 | 51.9306 | H-bond: Arg136, C-H bond: Glu50, Phe100 alkyl: Lys99 | **62.1958** | C-H bond: Pro770, Alkyl: Leu694, Leu768, Cys773, Val702, Lys721 |
| 33 | 38.5322 | Pipi-stacked: His75, alkyl: His82, Val74, His75 | 58.615 | H bond: Lys99, C-HbondLArg76, Alkyl: Ala86, Ala47, Pro79, Val43, Val167 | **54.1347** | H bond: Met769, C-H bond: Asp831, Pro770, Gln767, alkyl: Val702, Leu820, Ala719 |
| 34 | 31.5565 | alkyl: His82, His75, Val74, Leu438 | 40.1504 | Alkyl: Pro79, Ala86, Val120 | 40.7659 | H-bond: Lys721, C-H bond: Glu738, Alkyl: Leu820, Val702, Met742 |
| 35 | 25.912 | H bond: His75, C-H bond: Tyr407, Pipi stacked: Phe78, alkyl: His82 | 37.4363 | H-bond: Arg136, Amide Pi-stacked: Asn46, alkyl; Pro79, Ile78 | 34.1575 | Pi-cation: Lys721, Alkyl: Cys773, Leu820, Val702 |
| 36 | 26.6679 | alkyl: Phe78, His75, His82 | 37.8882 | Pi-cation: Lys99, alkyl: Val167, Ile90, Pro79, C-H bond: Asp101, Ala86 | 37.2819 | Alkyl: Lys721 |
| 37 | 27.1872 | C-H bond: Tyr407, Alkyl: Val74, His82 | 36.1046 | alkyl: Val43, Val167, Ala47, Ile78 | 36.107 | Pi-cation: Lys721, C-H bonds: Leu764, Thr766, Alkyl: Ala719, Val702, Leu820 |
| 38 | 29.6795 | C-H bond: Tyr407, Pipi-T shaped: Phe78, alkyl: His82, His75, Val74, Leu438 | 40.3901 | H-bond: Arg136, Pi-sigma: Asn46, C-H bond Val43, Arg46 alkyl: Lys99, Pro79, Ile78 | 41.5708 | Pi-sulphur: Met742, C-H bond: Glu738, Thr766, alkyl: Lys721, L eu820, Leu694, Ala719 |
| 39 | 24.4955 | H-bond: His75, C-H bond: His82, alkyl: Phe78, Val74 | 32.5458 | C-H bond: Lys99, Asn46, alkyl: Ile78, Pro79 | 34.6652 | H-bond: Cys773, C-H bond: Met769, Gly772, Alkyl: Leu820, Ala719 |
| 40 | **42.0383** | **H bond: His75, C-H bond: Glu77, His82, Val74, Pipi-stacked: Phe78, alkyl; Leu438, Trp70** | 46.8118 | Alkyl: Ala86, Pro79, Ile78, Ile90, Val43, Val167 | 44.2166 | Pi-sulphur: Cys773, C-h bond: Pro770, Met764, Gly772, alkyl: Leu694, Leu820, Val702, Ala719, Lys721 |
| 41 | 31.6004 | C-H bond: His82, Pipi-T shaped: Phe78, alkyl: Tyr407, Leu438, Val74, Trp70, | 41.9123 | H bond: Arg136, C-H bond: Arg76, Alkyl: Pro79, Ile78, Val167 | 43.9427 | H-bond: Lys721, C-H bond: Asp831, alkyl: Leu694, Leu820, Val702, Ala719 |
| 42 | 21.1282 | H-bonds: His75, C-H bonds: Asn71, alkyl: Val74, Phe78 | 27.0081 | H-bond: Arg136, C-H bond: Arg76, Pro79, Lys99, Glu50 | 24.5226 | H bond: Met764, C-H bond: Asp831, Alkyl: Ala719, Leu820, Val702 |
| 43 | 38.9445 | H-bonds: His75, C-H bond: His82, Alkyl: Val74, Phe78, Trp70, Leu438 | 48.963 | Alkyl: Val167, Ala47, Pro79, Ala86 | 48.4545 | Pi-sulphur: Met742, C-H bond: Glu738, Thr830, Thr766, Alkyl: Leu694, Leu820, Ala719, Cys773, Lys721 |
| 44 | 27.1162 | C-H bond: His75, Pipi-stacked: Phe78, His82, | 38.5292 | H-bond: Val43, Pi-cation: Lys99, Alkyl: Pro99, Ala47 | 36.4953 | H-bond: Pro770, Met742, Alkyl: Leu694 |
| 45 | 19.7682 | C-H bond: His82, Tyr407, Pipi-stacked: Phe78, alkyl:  : His75 | 26.5612 | C-H bond: Lys99, Asp101, Pi-sigma; Ile78 | 21.8908 | H-bond: Thr766, C-H bond: Gln767, Leu694, |
| 46 | 33.575 | alkylPhe78, Trp70, Val74 | 43.3544 | Alkyl: Ile78, Pro79 | 44.1085 | alkyl: Leu694, Leu820, Val702, Cys773, Lys721 |
| 47 | 35.8623 | alkyl: Val74, Trp70, Phe78 | 46.9146 | H-bond: Arg76, alkyl: Ile78, Pro79, Val167 | 40.6213 | alkyl: Leu694, Val702, Cys773, Lys721 |
| 48 | 29.8283 | alkyl: His75, Val74, Phe78, Tyr407, His82 | 43.0791 | alkyl: Val71, Val43, Val167, Ile78, Lys99 | 37.0215 | alkyl: Leu764, Leu820, Val702, Met742, Lys721 |
| 49 | 20.597 | H-bond: His82 | 39.5471 | H-bond: Arg76, Asp49, Asn46, C-H bond: Lys99 | 33.6329 | H-bond: Cys773, Met769, C-H bond: Gly772 |
| 50 | 26.4066 | C-H bond: Phe78, Tyr407, alkyl: Val74, His75, His82 | 39.5471 | Alkyl: Ala47, Ile90, Ile78, Ala86, Pro79 | 27.3125 | alkyl: Leu820, Ala719, Cys773 |
| 51 | 28.5223 | C-H bond: Tyr407, Asn71, alkyl: His82, Val74, Pipi-stacked: Phe78, PipiTshapedHis75 | 39.1841 | Pi-sigma: Asn46, C-H bond: Pro79, Alkyl; Ala47, Ile78 | 37.5019 | H-bond: Met769, C-H bond: Thr766, Leu768, alkyl: Lys721, Leu820, Ala719 |
| 52 | FD |  | FD |  | FD |  |
| 53 | 32.6782 | H-bond: His75, C-H bond: His82, alkyl: Met79, Val74, Trp70, Pi Pi-stacked: Phe78 | 47.0951 | H-bond: Arg136, C-H bonds: Asp101, Arg76, Asp73, alkyl; Val167, Val120, Ile78, | 46.1025 | H-bond: Met769, C-H bond: Glu738, alkyl: Met742, Lys721, Ala719 |
| 54 | 21.0181 | H-bond: His82, C-H bond: Tyr407, His75, Pi Pi-stacked and Pi Pi T-shaped: Phe78, alkyl: Val74 | 38.3045 | H-bonds: Asn46, Phe100, Alkyl: Ile78, val120, Ile90, Lys99, Pro79 | 33.9352 | H-bond: Met769, Thr766, C-H bond: Gly772, Asp831, alkyl: Val702, Ala719, Cys773, Leu820, Leu694 |
| 55 | 37.4934 | alkyl: His82, His75, Val74, Leu438, Trp70, Phe78 | 48.348 | Alkyl: Ala86, Ile90, Ile78, Val71, Val167 | 39.9267 | C-H bond: Pro770, Alkyl: Leu764, Cys773, Val702, Lys721, Met742 |
| 56 | 25.9003 | alkyl: Val74, His82, C-H bond: His75, Phe78 | 36.5635 | H-bond: Arg136, Alkyl: Pro79, Ile78, Ile90, Lys99 | 33.5103 | H-bond: Cys773, Thr830, C-H bond: Gly772, alkyl: Val702, Leu820, Leu694, Ala719 |
| 57 | 26.5539 | H bond: Asn71, C-H bond: Tyr407, Pipi-stacked: His82, alkyl: His82, Trp70, Val74 | 36.3527 | H-bond: Gly77, Asn46, alkyl: Ile78, Ile90, Lys99, Pro79 | 35.4201 | C-H bond: Pro770, alkyl: Val702, Leu820, Leu694, Ala719, Lys721 |
| 58 | 25.9589 | C-H bond: His75, His82, Pipi stacked: Phe78, alkyl: Val74 | 36.5069 | C-H bond: Lys99, Alkyl: Pro79, Ile90, Ile78 | 35.373 | Pi-cation: Lys721, Met742, C-H bond: Leu764, Ala719, Thr766, alkyl: Leu820, Leu694, Val702 |
| 59 | 27.0739 | H-bond: Asn71, C-H bond: Tyr407, alkyl: Val74, His82, His75, Phe78 | 33.6443 | Pi-cation: Lys99, C-H bond: Glu50, Ile78, Pro79, alkyl; Ile90, Ala86 | FD |  |
| HXP xanthene | 28.6446 | C-H bond: His75, Pipi stacked: Phe78, Alkyl: Val74 |  |  |  |  |
| Ascorbic acid | 25.3181 | H-bond: Asn71, C-H bond: His75 |  |  |  |  |
| clorobiocin |  |  | 69.9034 | H bond: Arg136, Asn46, Asp101, Pi-cation: Arg46, alkyl: Pro79, Lys99, Val71, Ala47 |  |  |
| Ampicillin |  |  | 49.407 | H-bond: Asn46, C-H bond: Lys99, Ile78, Pro79, Pi-cation: Asp73, Glu50, Asp49 |  |  |
| Erlotinib |  |  |  |  | 47.3483 | C-H bond: Pro770, Asp81, Alkyl: Cys773 |
| Doxorubcin |  |  |  |  | FD |  |

**Table S2: free binding energy values and molecular interactions of docked compounds in the three selected enzymes, XAN, 1KZN, and 1m17**
