# Supplementary material for: Green synthesis of Piper nigrum copper-based nanoparticles: in silico study and ADMET analysis to assess their antioxidant, antibacterial, and cytotoxic effects
Source: Front Chem. 2023 Sep 5;11:1218588. doi: 10.3389/fchem.2023.1218588 (PMC10509375; doi:10.3389/fchem.2023.1218588)
Supplement: Supplementary file 2 [file DataSheet1.docx]

**Supplementary Material**

**Green Synthesis of *Piper nigrum* Copper-based Nanoparticles: *In-Silico* Study and ADMET Analysis to Assess Their Antioxidant, Antibacterial, and Cytotoxic Effects**

**Modumudi Kiranmayee ^a^, Nambi Rajesh^a^, M. Vidya Vani^a^, Habeeb Khadri^b^, Arifullah Mohammed^c^, Suresh V. Chinni^d,e^, Gobinath Ramachawolran^f*^, Khateef Riazunnisa^a*^, Ashaimaa Y. Moussa^g*^**

^a^Department Biotechnology and Bioinformatics, Yogi Vemana University Kadapa, Andhra Pradesh, India-516005

^b^Department of Medical Laboratories, College of Applied Medical Sciences, Qassim University, Qassim 51452, Kingdom of Saudi Arabia.

^c^Department of Agriculture Science, Faculty of Agro-Based Industry, Universiti Malaysia Kelantan,17600Jeli, Kelantan, Malaysia.

^d^Department of Biochemistry, Faculty of Medicine, Bioscience, and Nursing, MAHSA University, 42610 Jenjarom, Selangor, Malaysia

^e^Department of Periodontics, Saveetha Dental College and Hospitals, Saveetha Institute of Medical and Technical Sciences, Chennai, India

^f^Department of Foundation, RCSI & UCD Malaysia Campus, No 4, Jalan Sepoy Lines, 10450 Georgetown, Pulau Pinang, Malaysia.

^g^Department of Pharmacognosy, Faculty of Pharmacy, Ain shams University, Cairo, Egypt, 11566

*****Corresponding Authors1: Ashaimaa Y. Moussa, [Ashaimaa_yehia@pharma.asu.edu.eg](mailto:Ashaimaa_yehia@pharma.asu.edu.eg) (AYM)

*Corresponding Author 2: Khateef Riazunnisa, [khateefriaz@gmail.com](mailto:khateefriaz@gmail.com) (KR)

* Corresponding Author 3: Gobinath Ramachawolran, [r.gobinath@rcsiucd.edu.my](mailto:r.gobinath@rcsiucd.edu.my)


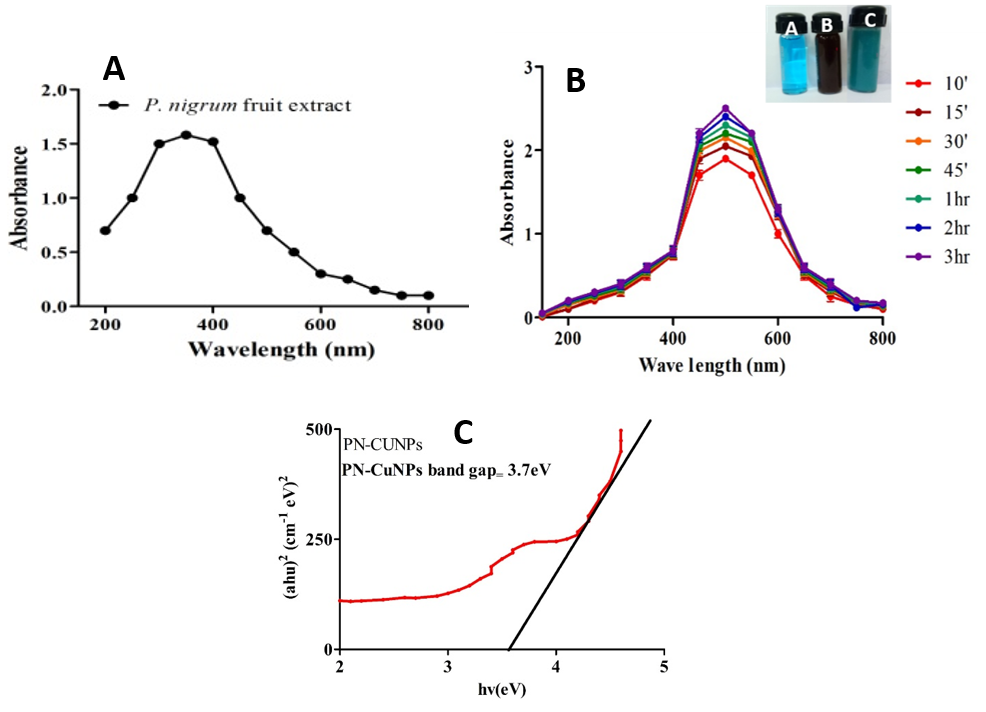


**Fig. S1** UV visible spectrum of (A) *P. nigrum* fruit extract (B) PN-CuNPs and (C) Band gap. [Inset: color change from brown to blue (A) Copper acetate (B) *P. nigrum* fruits extract (C) PN-CuNPs].


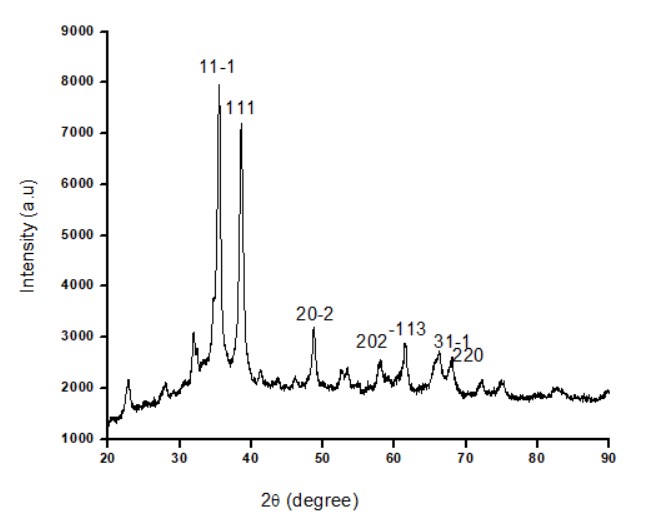


**Fig. S2** XRD analysis of PN-CuNPs


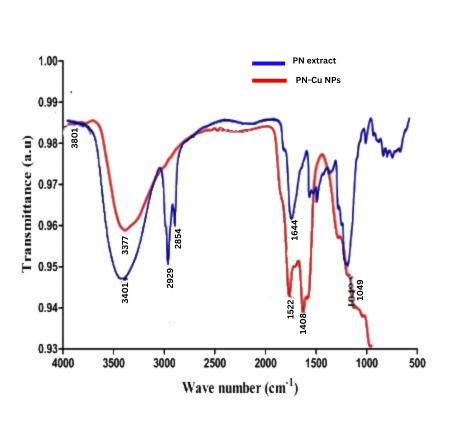


**Fig. S3** FTIR spectrum of *Piper nigrum* fruit extract and PN-CuNPs.


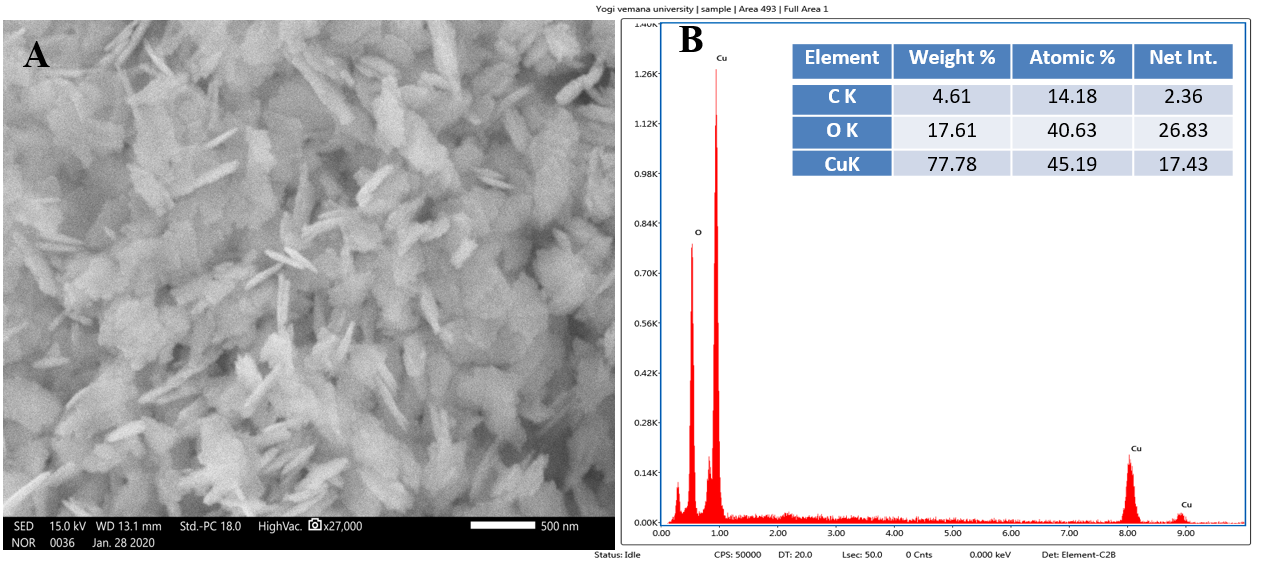


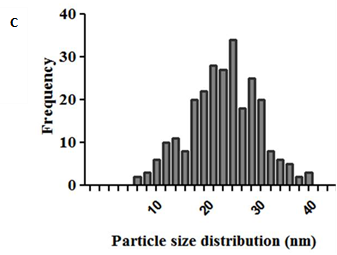


**Fig. S4** (A) SEM image (B) EDX analysis of PN-CuNPs (C) Particle size. Inset figure illustrates the elemental composition of nanoparticles.

**
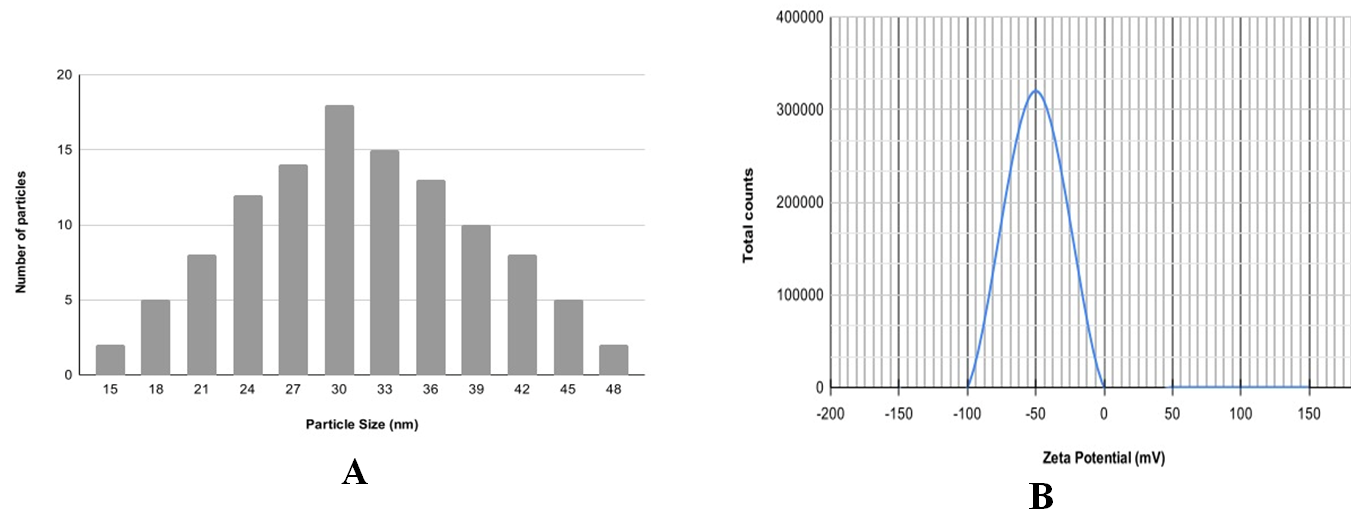
**

**Fig. S5 (A) Particle size distribution and (B) zeta potential of PN-CuNPs**

**
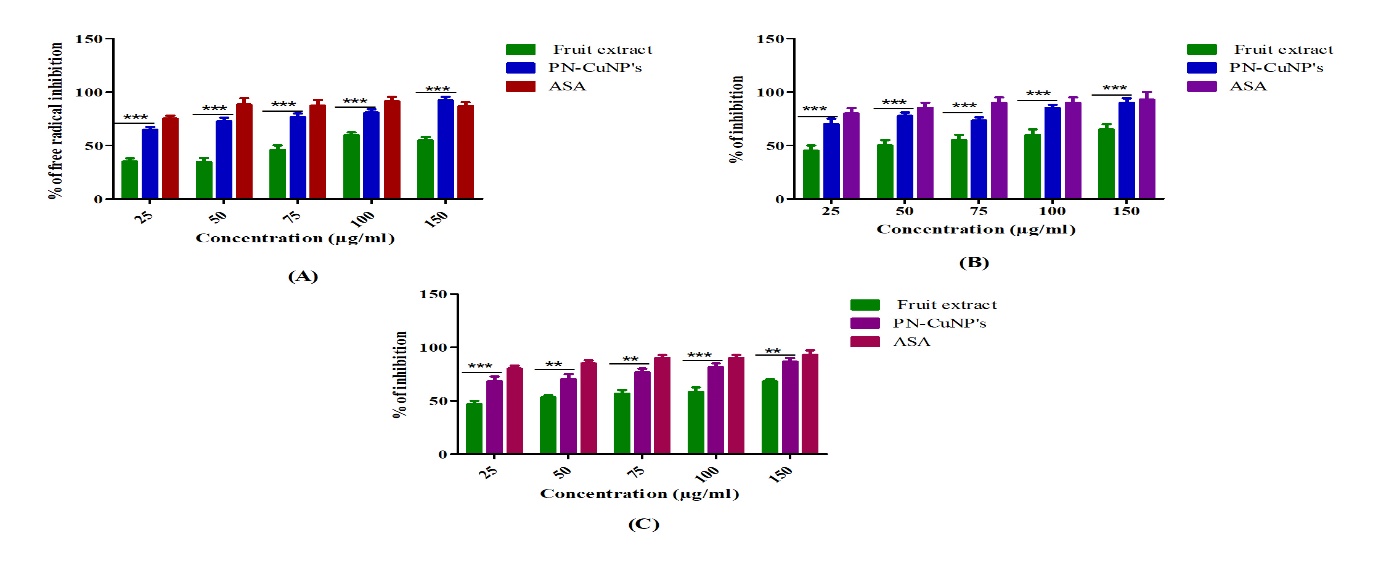
**

**Fig. S6** Antioxidant activity of *P. nigrum* fruits extract and PN-CuNPs. (A) DPPH free radical scavenging activity of *P. nigrum* fruits extract and PN-CuNPs (B) Hydroxy radical scavenging activity of *P. nigrum* fruits extract and PN-CuNPs. (C) Total antioxidant activity of *P. nigrum* fruits extract and PN-CuNPs. All experiments were run in triplicates and values are expressed as mean ± SEM. PN-CuNPs; *P. nigrum* copper nanoparticles. ASA- Ascorbic acid (Positive control).


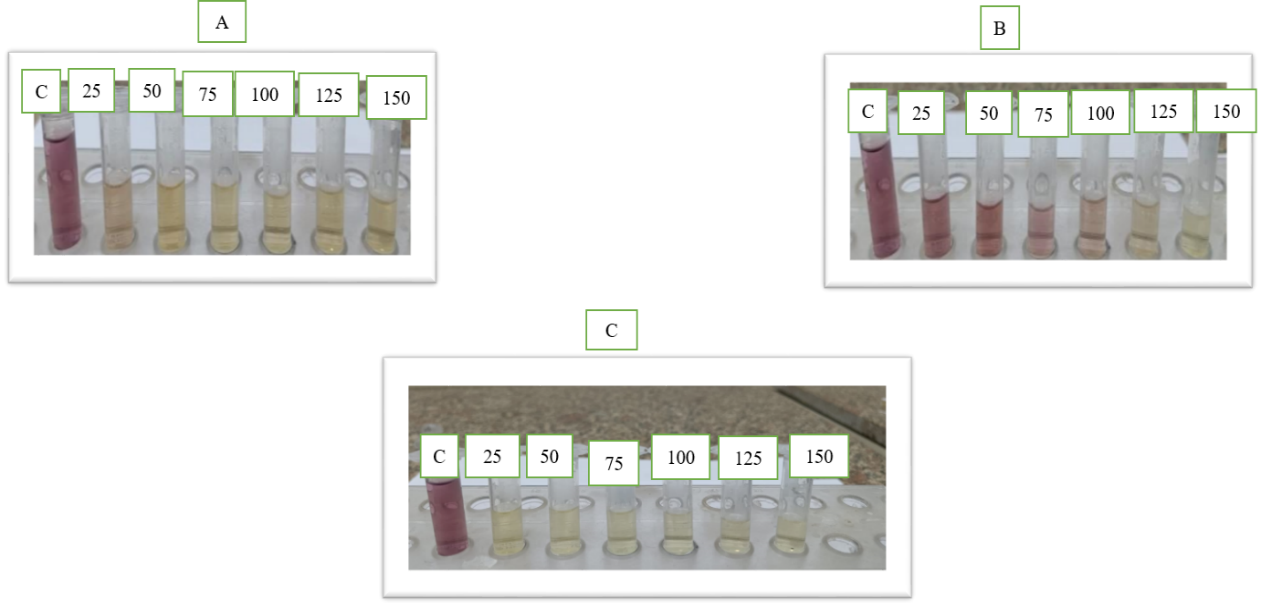


**Fig. S7** **­^_^** Colour change of DPPH free radical scavenging activity of *P. nigrum* fruits extract and PN-CuNPs at various concentrations ranging from 25-150 µg/ml (a) PN-CuNPs (b) *P. nigrum* fruits extract (c) Ascorbic acid (positive control); C- Positive Control (Ascorbic acid).

**
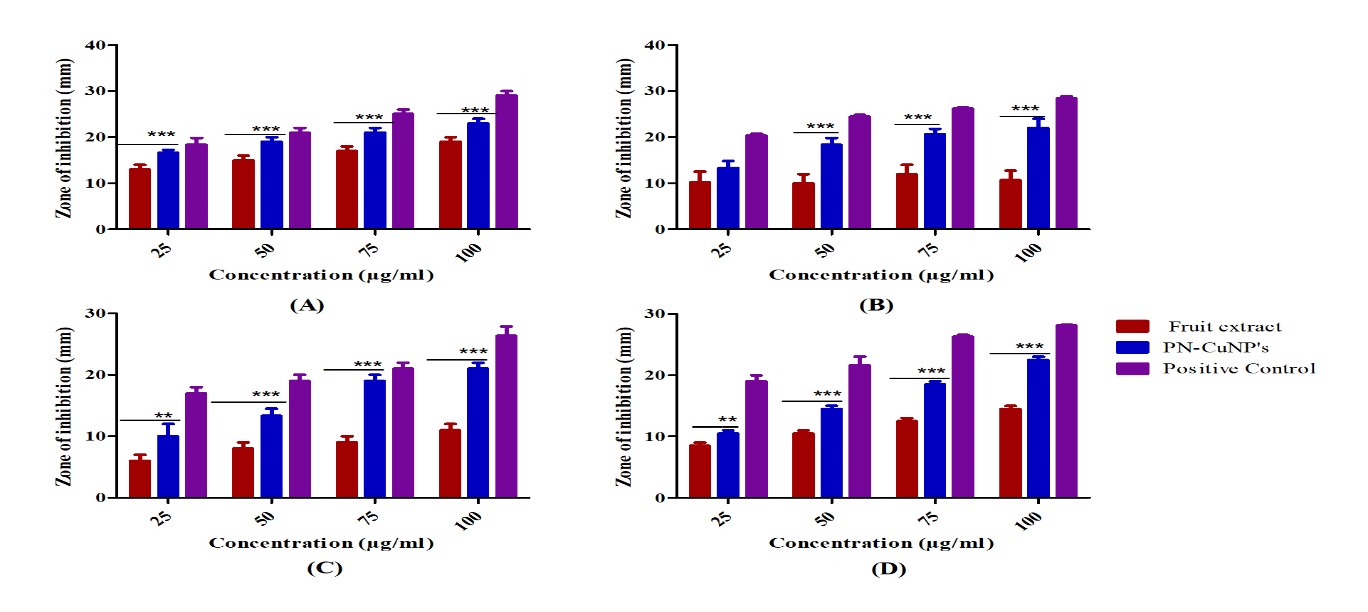
**

**Fig. S8** Antibacterial activity of *P. nigrum* fruits extract and PN-CuNPs along with positive control (Ampicillin). A. *Staphylococcus* *aureus* B. *Bacillus* *subtilis*; C. *Escherichia* *coli* and D. *Proteus* *vulgaris*. The data in all figures was measured in at least 3 samples the mean of three replicates S.D. are shown. Statistical analysis was done using TWO WAY ANOVA. ∗ Denotes significant difference of positive control with test samples at *P <* 0.001.

**
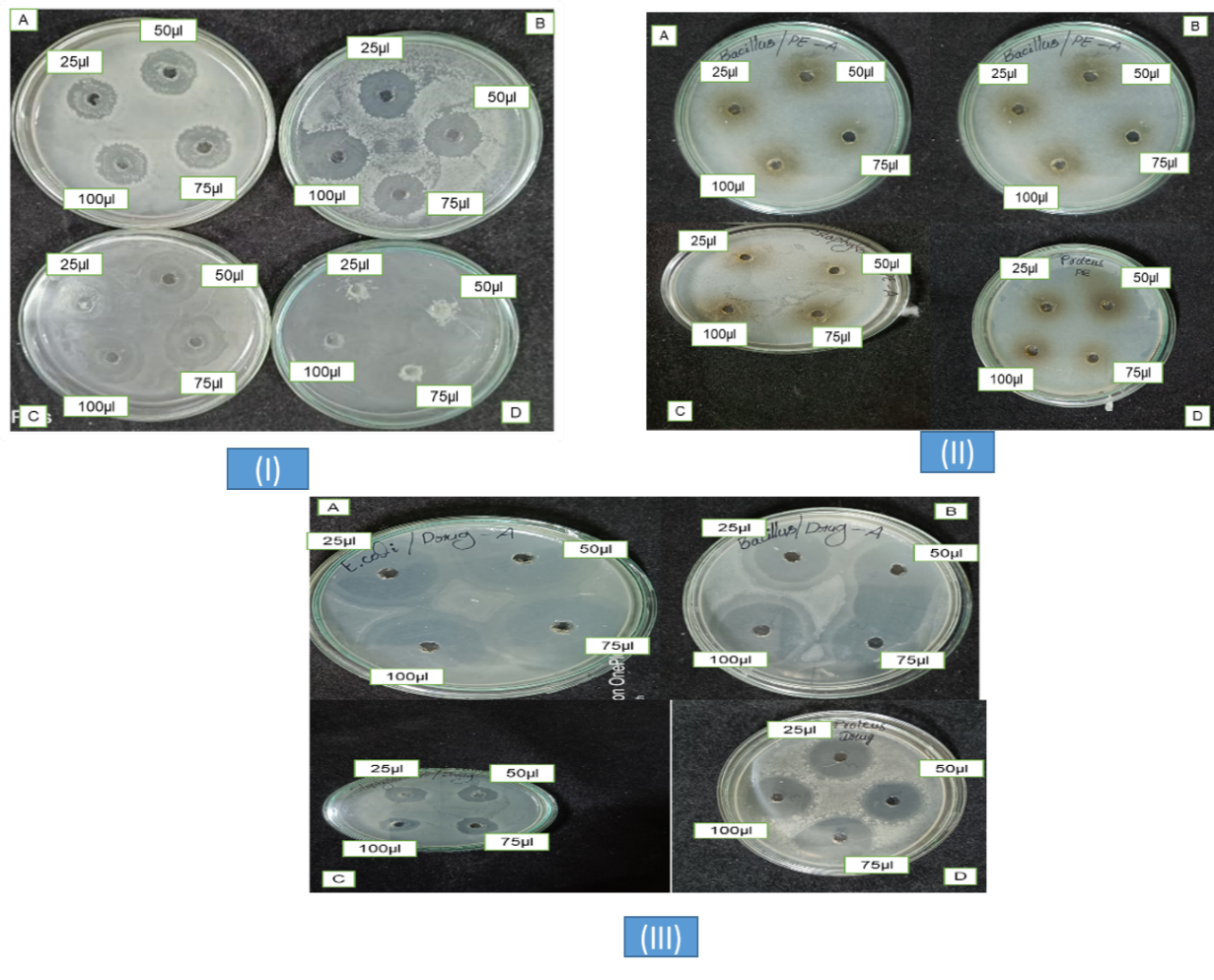
**

**Fig. S9 ^_^** Antibacterial zone of inhibition of *P. nigrum* fruits extract and PN-CuNPs along with positive control (Ampicillin) at various concentration ranging from 25-100 µg/ml. (I) Antibacterial activity of PN-CuNPs. (II) Antibacterial activity of *P. nigrum* fruits extract (III) Antibacterial activity of Positive control (Ampicillin). A. *Staphylococcus* *aureus* B. *Bacillus* *subtilis*; C. *Escherichia* *coli*; D. *Proteus* *vulgaris.*


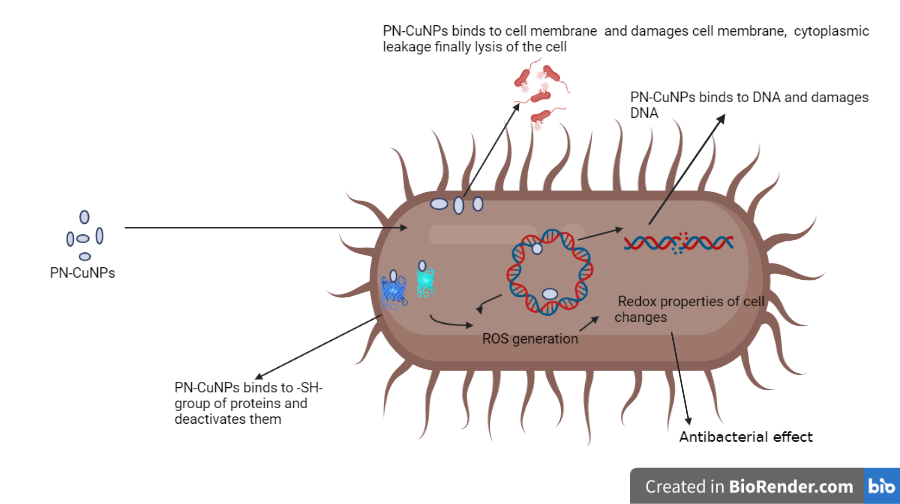


**Fig. S10** Proposed mechanism of antibacterial activity of PN-CuNPs against pathogenic bacteria.


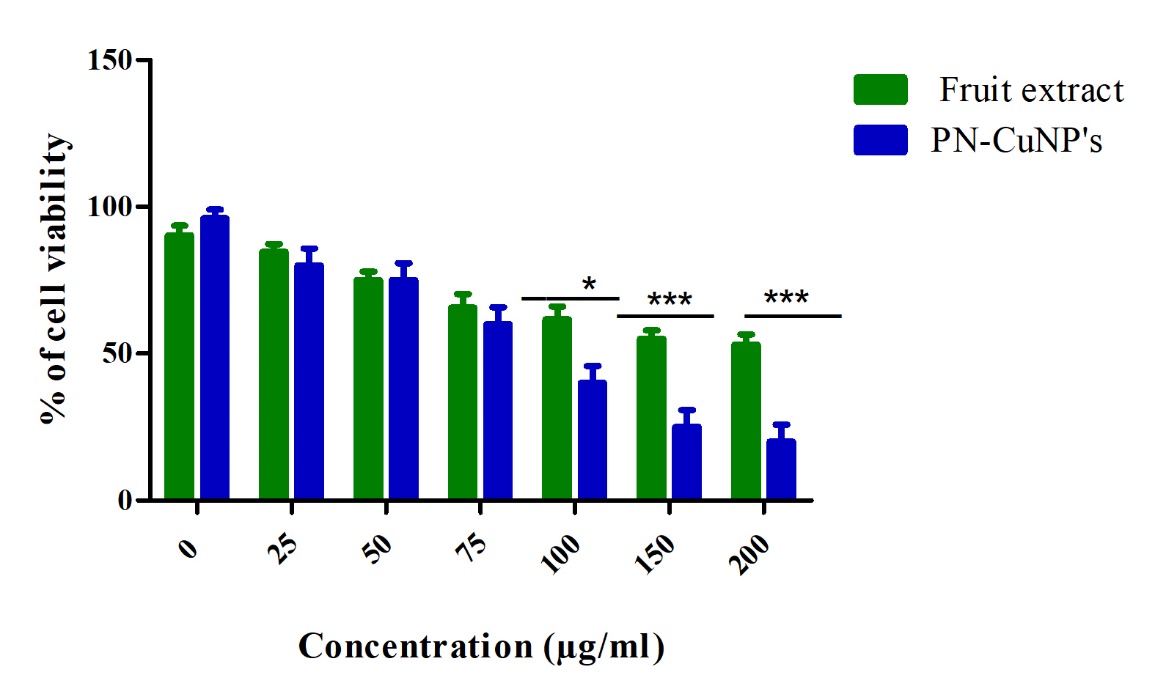


**Fig. S11** Cytotoxicity activity of *P. nigrum* fruits extract and PN-CuNPs against MCF-7 cancerous cell lines. Data expressed as mean ± SD (n = 3). *** Indicates a significant increase relative to the untreated control (p < 0.001).


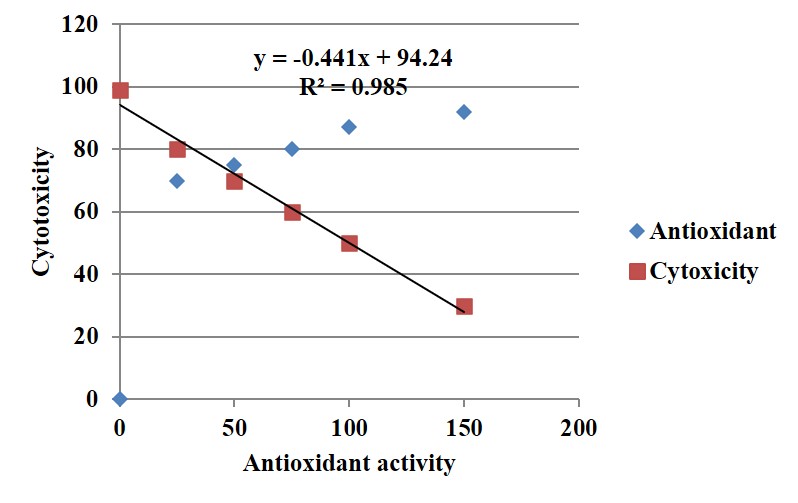


**Fig. S12** Correlation between the antioxidant and cytotoxic activity of PN-CuNPs by linear regression analysis.

**
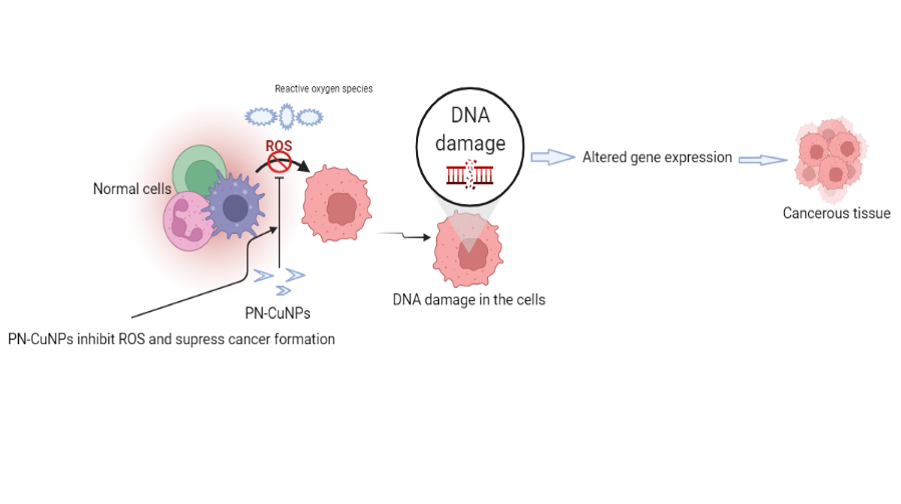
**

**Fig. S13** Proposed mechanism of cytotoxicity of PN-CuNPs against MCF-7 cancerous cell lines.

**Table S1.** FT-IR analysis and probable functional groups of PN-CuNPs

| **PN extract wave numbers (cm^-1^)** | **PN-CuNPs wave number (cm^-1^)** | **Probable functional group** | **Compound Class** |
| --- | --- | --- | --- |
| 3801 | 3801 | O-H strech | Alcohol |
| 3401 | 3377 | N-H stretch | Primary amine |
| 2929 | 2929 | C-H stretch | Alkane |
| 2854 | 2854 | N-H stretch | Amine |
| 1644 | 1644 | C=C stretch | Alkene |
| 1522 | 1522 | C–H, C=C | Aromatic |
| 1408 | 1408 | O-H stretch | Alcohol |
| 1049 | 1049 | CO-O-CO stretch | Anhydride |

**Supplementary legends**

**Figure S1:** Colour change of DPPH free radical scavenging activity of *P. nigrum* fruits extract and PN-CuNPs at various concentrations ranging from 25-150 µg/ml (a) PN-CuNPs (b) P. nigrum fruits extract (c) Ascorbic acid (positive control); C- Positive Control (Ascorbic acid).

**Figure S2**: Antibacterial zone of inhibition of *P. nigrum* fruits extract and PN-CuNPs along with positive control (Ampicillin) at various concentration ranging from 25-100 µg/ml. (I) Antibacterial activity of PN-CuNPs. (II) Antibacterial activity of *P. nigrum* fruits extract (III) Antibacterial activity of positive control (ampicillin). A. *Staphylococcus aureus* B. *Bacillus subtilis; C. Escherichia coli; D. Proteus vulgaris.*

**Figure S3**: *Piper nigrum* fruits and some main chemical structures adopted from Shityakov et al.

**Figure S4**: UV visible spectrum of (A) *P. nigrum* fruit extract (B) PN-CuNPs and (C) Band gap. [Inset: color change from brown to blue (A) Copper acetate (B) *P. nigrum* fruits extract (C) PN-CuNPs].

**Figure S5** XRD analysis of PN-CuNPs

**Figure S6** FTIR spectrum of (A) *P. nigrum* fruit extract (B) PN-CuNPs

**Figure S7** (A) SEM image (B) EDX analysis of PN-CuNPs (C) Particle size. Inset figure illustrates the elemental composition of nanoparticles.

**Figure S8** (A) Particle size distribution and (B) zeta potential of PN-CuNPs

**Figure S9** Antioxidant activity of *P. nigrum* fruits extract and PN-CuNPs. (A) DPPH free radical scavenging activity of *P. nigrum* fruits extract and PN-CuNPs (B) Hydroxy radical scavenging activity of *P. nigrum* fruits extract and PN-CuNPs. (C) Total antioxidant activity of *P. nigrum* fruits extract and PN-CuNPs. All experiments were run in triplicates and values are expressed as mean ± SEM. PN-CuNPs*; P. nigrum* copper nanoparticles. ASA- ascorbic acid (positive control).

**Figure S10** Antibacterial activity of *P. nigrum* fruits extract and PN-CuNPs along with positive control (ampicillin). A. *Staphylococcus aureus B. Bacillus subtilis; C. Escherichia coli and D. Proteus vulgaris.* The data in all figures was measured in at least 3 samples the mean of three replicates S.D. is shown. Statistical analysis was done using TWO WAY ANOVA. ∗ Denotes significant difference of positive control with test samples at P < 0.001.

**Figure S11** Proposed mechanism of antibacterial activity of PN-CuNPs against pathogenic bacteria.

**Figure S12** Cytotoxicity activity of *P. nigrum* fruits extract and PN-CuNPs against MCF-7 c

**Figure S13** Proposed mechanism of cytotoxicity of PN-CuNPs against MCF-7 cancerous cell lines. Data expressed as mean ± SD (n = 3). *** Indicates a significant increase relative to the untreated control (p < 0.001).
